# GEM: scalable and flexible gene–environment interaction analysis in millions of samples

**DOI:** 10.1093/bioinformatics/btab223

**Published:** 2021-05-25

**Authors:** Kenneth E Westerman, Duy T Pham, Liang Hong, Ye Chen, Magdalena Sevilla-González, Yun Ju Sung, Yan V Sun, Alanna C Morrison, Han Chen, Alisa K Manning

**Affiliations:** Department of Medicine, Clinical and Translational Epidemiology Unit, Mongan Institute, Massachusetts General Hospital, Boston, MA 02114, USA; Metabolism Program, Broad Institute of MIT and Harvard, Cambridge, MA 02142, USA; Department of Medicine, Harvard Medical School, Boston, MA 02115, USA; Human Genetics Center, Department of Epidemiology, Human Genetics and Environmental Sciences, School of Public Health, The University of Texas Health Science Center at Houston, Houston, TX 77030, USA; Human Genetics Center, Department of Epidemiology, Human Genetics and Environmental Sciences, School of Public Health, The University of Texas Health Science Center at Houston, Houston, TX 77030, USA; Department of Medicine, Clinical and Translational Epidemiology Unit, Mongan Institute, Massachusetts General Hospital, Boston, MA 02114, USA; Department of Medicine, Clinical and Translational Epidemiology Unit, Mongan Institute, Massachusetts General Hospital, Boston, MA 02114, USA; Metabolism Program, Broad Institute of MIT and Harvard, Cambridge, MA 02142, USA; Department of Medicine, Harvard Medical School, Boston, MA 02115, USA; Department of Psychiatry, Washington University School of Medicine, St. Louis, MO 63130, USA; Division of Biostatistics, Washington University School of Medicine, St. Louis, MO 63130, USA; Department of Epidemiology, Emory University Rollins School of Public Health, Atlanta, GA 30322, USA; Department of Biomedical Informatics, Emory University School of Medicine, Atlanta, GA 30322, USA; Human Genetics Center, Department of Epidemiology, Human Genetics and Environmental Sciences, School of Public Health, The University of Texas Health Science Center at Houston, Houston, TX 77030, USA; Human Genetics Center, Department of Epidemiology, Human Genetics and Environmental Sciences, School of Public Health, The University of Texas Health Science Center at Houston, Houston, TX 77030, USA; Center for Precision Health, School of Public Health and School of Biomedical Informatics, The University of Texas Health Science Center at Houston, Houston, TX 77030, USA; Department of Medicine, Clinical and Translational Epidemiology Unit, Mongan Institute, Massachusetts General Hospital, Boston, MA 02114, USA; Metabolism Program, Broad Institute of MIT and Harvard, Cambridge, MA 02142, USA; Department of Medicine, Harvard Medical School, Boston, MA 02115, USA

## Abstract

**Motivation:**

Gene–environment interaction (GEI) studies are a general framework that can be used to identify genetic variants that modify the effects of environmental, physiological, lifestyle or treatment effects on complex traits. Moreover, accounting for GEIs can enhance our understanding of the genetic architecture of complex diseases and traits. However, commonly used statistical software programs for GEI studies are either not applicable to testing certain types of GEI hypotheses or have not been optimized for use in large samples.

**Results:**

Here, we develop a new software program, GEM (Gene–Environment interaction analysis in Millions of samples), which supports the inclusion of multiple GEI terms, adjustment for GEI covariates and robust inference, while allowing multi-threading to reduce computation time. GEM can conduct GEI tests as well as joint tests of genetic main and interaction effects for both continuous and binary phenotypes. Through simulations, we demonstrate that GEM scales to millions of samples while addressing limitations of existing software programs. We additionally conduct a gene-sex interaction analysis on waist-hip ratio in 352 768 unrelated individuals from the UK Biobank, identifying 24 novel loci in the joint test that have not previously been reported in combined or sex-specific analyses. Our results demonstrate that GEM can facilitate the next generation of large-scale GEI studies and help advance our understanding of the genetic architecture of complex diseases and traits.

**Availability and implementation:**

GEM is freely available as an open source project at https://github.com/large-scale-gxe-methods/GEM.

**Supplementary information:**

Supplementary data are available at *Bioinformatics* online.

## 1 Introduction

Genome-wide association studies (GWAS) have successfully led to many discoveries that associate genetic alleles with complex human diseases. However, many complex diseases are also influenced by non-genetic risk factors, such as lifestyle habits (e.g. cigarette smoking), environmental exposures (e.g. toxin), physiological effects (e.g. obesity) or treatment interventions (e.g. daily aspirin therapy). Most GWAS do not address the scientific question regarding how genetic effects on complex diseases may depend on these non-genetic factors. Gene–environment interaction (GEI) studies characterize the interplay of genetic and non-genetic effects on complex diseases. GEI models can further identify genetic variants with heterogeneous effects in different age, sex/gender or race/ethnic groups, improving our understanding of the genetic architecture of complex diseases and traits in the context of non-genetic risk factors that contribute to health disparities.

Genomic datasets with millions of study participants are now being collected at an unprecedented scale: examples include the *All of Us* Research Program (anticipated *N* ∼ 1 000 000), the U.K. Biobank (*N* ∼ 500 000) and the Million Veteran Program (MVP, *N* ≥ 1 000 000). While such big genomic data provide excellent opportunities for GEI studies, tools for GEI analyses have remained static and have not adapted to the needs of modern epidemiological investigations. Several challenges to GEI studies were articulated in a recent review (Gauderman *et al.*, 2017), including the lack of power in GEI tests and lack of tools for large-scale, ‘omics-driven’ data. The emergence of exposure data from the environmental ‘exposome’ (Rappaport, 2011; Wild, 2005), methylation studies, metabolomic profiling in blood and other tissues and microbiome studies (collectively, ‘Omics exposures’) offer another axis to explore the interplay between genetic variants and complex diseases, especially in models that consider interactions with multiple exposures (Kim *et al.*, 2019). Furthermore, GEI effects can be biased if epidemiological confounders of the interaction effects are not properly controlled (Keller, 2014). The bias can be corrected, and in fact the relationships between multiple factors can be characterized, by including interaction terms for multiple exposures and covariates in the model (Keller, 2014). Another issue with these various continuous environmental and ‘Omics exposures’ is that model-based GEI inference is invalid when linear-model assumptions are violated (when the exposure has a non-linear relationship with the outcome and/or residual variance is not constant). Robust standard errors have been proposed as a remedy measure (Voorman *et al.*, 2011), but efficient analytical tools are still needed to scale up robust inference procedures to large samples.

Multiple existing software programs enable genome-wide GEI testing (Aulchenko *et al.*, 2010; Kutalik *et al.*, 2011; Chang *et al.*, 2015; Lin *et al.*, 2014; Bi *et al.*, 2019; Bhattacharjee *et al.*, 2012; Gauderman *et al.*, 2016). Many are not optimized for analysis in hundreds of thousands or millions of individuals, with prohibitive computational resource requirements and lacking multi-threading capability to decrease runtimes. QUICKTEST, PLINK2, CGEN and GxEScan each enable efficient analysis in large samples, but none allow for robust inference and multiple interaction terms for both continuous and binary outcomes. The authors of SPAGE recently introduced a matrix projection algorithm for interaction testing, and showed that this combined with a score test improved runtimes compared to the standard Wald test, as is used in the named programs above (Bi *et al.*, 2019). However, SPAGE currently analyzes only binary traits, without robust standard errors or multi-threading capabilities.

In an effort to enable biobank-scale analysis while supporting optimal and flexible statistical inference, we have developed an open-source software tool called GEM (Gene–Environment interaction analysis in Millions of samples). Our tool leverages a matrix projection algorithm to conduct approximate inference while accommodating important additional capabilities including robust standard errors and multiple GEI terms. The aims of this paper are to introduce the GEM methodology, benchmark GEM against existing software tools for GEI and demonstrate gains in computational efficiency from both the GEM method and our multi-threading software implementation, and finally demonstrate the use of GEM in a biobank-scale, genome-wide interaction study of waist-hip ratio, an anthropometric trait with known differences in genetic architecture between men and women (Pulit *et al.*, 2019).

## 2 Materials and methods

### 2.1 GEM model

Briefly, GEM considers a generalized linear model for unrelated individuals:

$$g\left(\mu_{i}\right)=X_{i}\beta_{X}+G_{i}\beta_{G}+C_{i}\beta_{C}+S_{i}\beta_{S},$$

where $\mu_{i}=E(Y_{i}|X_{i},G_{i})$ is the conditional mean of the phenotype $Y_{i}$ for individual i given covariates $X_{i}$ (including an intercept for the model) and the genotype $G_{i}$ of a single genetic variant. We consider two sets of gene–environment interaction terms: (i) *c* gene–environment interaction covariates $C_{i}$ not tested in our genome-wide scan, which are the product between $G_{i}$ and a subset of $X_{i}$, and are important for adjusting for potential confounding effects (Keller, 2014); and (ii) q gene–environment interaction terms of interest $S_{i}$, which are _the product of_  $G_{i}$ and another subset of $X_{i}$. The link function g(·) is a monotone function (usually the identity link function for continuous phenotypes, and the logit link function for binary phenotypes).

The GEM method is described in detail in Supplementary Methods. Through projection of the gene–environment interaction matrix onto the genetic matrix, the GEM method greatly reduces repeated calculations in fitting these separate models (e.g. adjusting for the same covariates repeatedly) and scales linearly with the sample size. Robust standard errors are optionally included in the calculation of standard errors for marginal genetic effects and GEI effects. Asymptotic *P*-values are calculated for each variant using adjusted score tests corresponding to the interaction effect (q degrees of freedom) or joint genetic effects (q+1 degrees of freedom).

### 2.2 Computational approach

The GEM software tool implements the GEM model with additional multi-threading via the C++ *Boost* library to parallelize our genome-wide scan across variants when multiple computing cores are available. Docker images and cloud-computing workflows written in Workflow Description Language (WDL) are distributed on github.com/large-scale-gxe-methods and the Dockstore platform (O’Connor *et al.*, 2017), providing GEM through a consistent interface across a variety of computational environments.

### 2.3 Alternative models and methods

Existing software programs that implement GEI for unrelated samples and common genetic variants were compared to GEM to establish the statistical validity of our method and compare performance: ProbABEL (Aulchenko *et al.*, 2010), QUICKTEST (version 0.95) (Kutalik *et al.*, 2011), PLINK2 (version alpha 2.3) (Chang *et al.*, 2015), SUGEN (version 8.11) (Lin *et al.*, 2014) and SPAGE (version 0.1.5) (Bi *et al.*, 2019). A comparison of the methods and software features available from these tools can be found in Table 1.

**Table 1.** Comparison of methods and software features implemented in GEI software tools

Tool	Version	Algorithmic approach	Multiple interactions	Interaction covariates	Robust SEs	Multithreading available
GEM	1.2	Matrix projection	Yes	Yes	Yes	Yes
ProbABEL	0.5.0	Classical linear/logistic model	No	No	Yes	No
QUICKTEST	0.95	Classical linear model	No	No	Yes	No
PLINK2	Alpha 2.3	Classical linear/logistic model	Yes	Yes	No	Yes
SUGEN	8.11	Classical linear/logistic model	Yes	No	Yes	No
SPAGE	0.1.5	Matrix projection	No	No	No	No

### 2.4 Simulated data for software benchmarking

For the purposes of comparing computational resource usage across software tools, a series of simulated genotype datasets were created with 100 000 variants and 6 different sample sizes: 10 000, 50 000, 100 000, 500 000, 1 million and 5 million. We simulated continuous and binary traits: a continuous outcome and a binary outcome with a case-control ratio of 1:3, one binary exposure and one binary covariate and 29 continuous covariates. Workflows implementing the GEI tests were run on the Analysis Commons hosted on the DNAnexus platform using the single exposure and all combinations of the following parameters: outcome type {binary, continuous}, number of non-exposure covariates {0, 3, 30} and sample size (as above). Benchmarking runs all used a single thread for computation. Runs that took longer than 7 days or used more than 100 GB of memory were terminated and noted in the results and figures.

GEM results were compared to those of ProbABEL to assess whether there were meaningful differences in their statistical output, given that ProbABEL is a commonly used program that does not use a matrix projection approach. Summary statistics were retrieved for a random subset of 5000 variants (corresponding to the 10 000-sample, robust, 3-covariate models) for both GEM and ProbABEL, and both interaction test *P*-values and main and interaction joint test *P*-values were compared.

### 2.5 UK Biobank Data for statistical simulations and real data example

UK Biobank is a cohort study containing deep phenotyping and genome-wide genetic data for approximately 500 000 participants living in the UK (Bycroft *et al.*, 2018). Imputed genotype data were pre-processed as described previously (Bycroft *et al.*, 2018) and accessed under UK Biobank projects 27892 and 42646. For this analysis, imputed genotypes (version 3) were filtered for minor allele frequency (MAF) > 0.005 and imputation INFO score > 0.5 based on the available variant annotation files from UK Biobank. Samples were filtered to include only those corresponding to unrelated individuals self-identifying as of white British ancestry who had not revoked consent for analysis, did not display sex chromosome aneuploidy, and had matching reported and genetically inferred sex. These filters left 352 768 UK Biobank study participants in the final dataset.

### 2.6 Power and type I error simulations

Phenotype simulations to evaluate power and type I error of the GEM method were carried out using R version 3.6.0 (R Core Team, 2019). A series of simulated phenotypes were generated from a subset of UK Biobank chromosome 21 and 22 imputed genotype dosages (a subset with MAF > 0.005). For type I error evaluation, three exposures were simulated (continuous, binary, log-normal), each having 10% variance explained by a random 100 variants from chromosome 21. Next, 185 continuous phenotypes were simulated for each of these exposures (185 phenotypes × 11.2 million = 2 billion *P*-values per exposure) to allow us to evaluate empirical type I error rates at genome-wide significance. Each phenotype was simulated to have 10% variance explained by the associated exposure and 10% variance explained by a random 100 variants from chromosome 22. In order to simulate exposure mis-specification, a fourth set of phenotypes was generated with the same amount of variance explained by a quadratic (rather than linear) effect of the continuous exposure. Genome-wide scans on ∼11.2 million variants for chromosomes 1–22 (MAF > 0.005) were run for each phenotype (4 settings × 185 exposures = 740 total runs) using both model-based and robust standard errors. Type I error was then evaluated at genome-wide significance for chromosomes 1–20 (for which no genetic effect of any kind was present). In addition, potential inflation was visualized via quantile-quantile plots for chromosome 21 (for which a gene–environment correlation was present for a subset of variants), and chromosome 22 (for which a genetic main effect was present for a subset of variants).

For power evaluation, a series of traits were generated based on a random set of 100 variants from chromosome 16. First, ten continuous exposures were simulated from a standard normal distribution. Next, ten normally distributed phenotypes were generated based on these variants and exposures for each combination of the following parameter values: $R_{G,\;\mathrm{total}}^{2}$ {0,10%}, $R_{E,\;\mathrm{total}}^{2}$ {10%}, $R_{G\times E,\;\mathrm{total}}^{2}$ {0.1%, 0.25%, 0.5%, 0.75%, 1%, 1.25%, 1.5%, 1.75%, 2%} and the number of exposures with GEI effects (**K**) {1, 2, 5, 10} (see Supplementary Table S1). As K was varied, GEI effects were distributed equally across the set of relevant exposures while retaining the same overall variance explained by GEIs. Finally, for each parameter combination, power was calculated as the proportion of tests reaching genome-wide significance ($\alpha =5\times 10^{-8}$) across the ten phenotypes (1000 tests in total per combination).

### 2.7 Application to waist–hip ratio in UK Biobank

To illustrate the performance of GEM for use in a biobank-scale, genome-wide interaction study, we conducted a gene-by-environment interaction study of the differences in waist-hip ratio (WHR) between men and women in unrelated, British-ancestry UK Biobank participants. We used sex (confirmed by genotype) as our exposure of interest. WHR was inverse-normal transformed prior to analysis. Additional covariates included age, age^2^, 10 genetic principal components (*PC1*-*PC10*), microarray used for genotyping (*array*) and body mass index (*BMI*):

$$\mathrm{WHR}\;∼\;G+\mathrm{sex}+\mathrm{age}+{\mathrm{age}}^{2}+\mathrm{BMI}+\mathrm{array}+PC1+\cdots +PC10+G\;\times \mathrm{sex}+G\times \mathrm{BMI}$$

The significance of the interaction effect was derived from the G×sex term and joint tests were conducted with the G and G×sex coefficient estimates. The G×BMI interaction covariate term controlled for potential confounding by *BMI* in this model. An additional analysis was performed in which age, coded as a binary variable of less than or greater than 50 years old, was added as a covariate and additional G×age interaction term. The resulting interaction effects (G×sex and G×age) were tested jointly using two degrees of freedom.

Results from the UK Biobank analysis were processed using the FUMA web tool (Watanabe *et al.*, 2017), which defines loci using linkage disequilibrium (LD). Variants with *P* < 5 × 10^−8^ were used as index variants, and additional variants with *P* < 0.05, within 250 kb of the index variant and having LD *r*^2^ > 0.2 with the index variant were merged into a single locus. We used this LD-based procedure to obtain the list of significant loci from the G×sex interaction test statistics and the joint test statistics. These loci were then compared to a recent genome-wide association study (GWAS) meta-analysis for BMI-adjusted WHR that included UK Biobank as one of the component datasets (Pulit *et al.*, 2019). Summary statistics from this analysis were retrieved (https://github.com/lindgrengroup/fatdistnGWAS; accessed April 13, 2020) for genome-wide significant marginal genetic effects in the entire sample, the female sample and the male sample. A subset of these variants were noted as having effect differences between males and females at a significance threshold adjusting for the number of index variants tested for interaction (P_GIANT; WHR; interaction_ < 3.3 × 10^−5^.) We re-derived these genome-wide tests for sex dimorphism using the EasyStrata R package (Winkler *et al.*, 2015). We assessed the value of the interaction approach implemented in GEM by annotating our significant loci with the presence or absence of the locus in the list of significant loci from Pulit *et al.* (2019). We extended each locus identified in our clumping procedure by 100 kb on each side and labeled each as novel if the region did not contain a variant with P_GIANT; WHR; interaction_ less than 5 × 10^−8^ or 3.3 × 10^−5^ from the Pulit *et al.* (2019) meta-analysis at either genome-wide or regional significance level, respectively. We similarly assessed the value of the joint test by expanding our loci by 100 kb and labeling them as novel if they did not contain a significant variant from the Pulit *et al.* (2019) summary statistics in either the entire sample meta-analysis, the female sample meta-analysis or the male sample meta-analysis.

To better understand the influence of including the G×BMI interaction covariate, we assessed which loci were significant (*P* < 5 × 10^−8^) in either the Pulit *et al.* (2019) meta-analysis or our analysis, but not both. For these variants, a second analysis was run with GEM without the G×BMI interaction covariate term and *P*-values at index variants for each of these loci were compared to those from the primary analysis.

To address concerns about genomic inflation, and considering prior conclusions that GEI analyses require approximately four times the sample size for equivalent power to a marginal genetic analysis of equal magnitude (Smith and Day, 1984; Thomas, 2010), the UK Biobank analysis was repeated three times after a random down-sampling to 87 695 individuals (24.859%, calculated as the product of the proportions of males and females in the dataset). Genomic inflation lambda values for the marginal genetic effects were calculated from GEM test statistics and compared to the primary (full-sample) GEM test statistics.

## 3 Results

### 3.1 Type 1 error and power of GEM

A series of genome-wide interaction scans were performed to assess type I error rates of the GEM algorithm. Using robust standard errors, no inflation of test statistics was observed (at significance level α = 5 × 10^−8^) for genetic variants on chromosomes 1-20 for which no genetic effects were simulated (Table 2). An inflated type I error rate was observed for both interaction and joint tests in the presence of a mis-specified (quadratic) environmental effect when robust standard errors were not used, in agreement with a known result (Voorman *et al.*, 2011). Supplementary Figures S1–S3 display quantile-quantile plots for these variants, as well as those for variants correlated with the exposure and with the phenotype. No unexpected inflation of *P*-values was observed for tests using robust standard errors other than in the context of both gene–environment correlation and a mis-specified exposure effect, as expected based on previous results (Zhang *et al.*, 2020).

**Table 2.** Type I error rates of GEM at α = 5 × 10^−8^

Simulated exposure distribution	Interaction test		Joint test	
	Type I error (model-based SEs)	Type I error (robust SEs)	Type I error (model-based SEs)	Type I error (robust SEs)
Binary exposure	3.14 × 10^-8^	3.04 × 10^-8^	3.93 × 10^-8^	3.93 × 10^-8^
Continuous exposure	3.58 × 10^-8^	3.53 × 10^-8^	4.38 × 10^-8^	4.28× 10^-8^
Log-normal exposure	4.98 × 10^-8^	3.33× 10^-8^	2.24× 10^-8^	2.64 × 10^-8^
Continuous exposure with quadratic effect	3.29 × 10^-6^	3.43 × 10^-8^	1.57× 10^-6^	2.44× 10^-8^

**Fig. 1.**
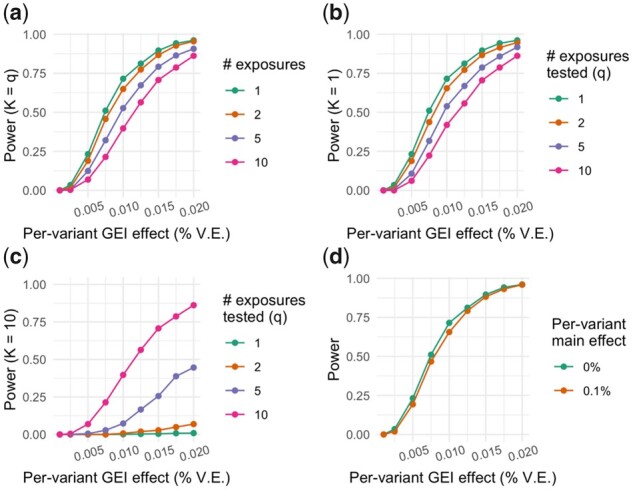
Power of the interaction test from the GEM method. Statistical power is shown on the *y*-axis reflecting the fraction of interaction tests with *P* < 5 × 10^−8^ (calculated based on 1000 tests). (**a**) Total interaction effect (*x*-axis), in terms of phenotypic variance explained, is partitioned equally among *K* exposures (*K* = 1, 2, 5 and 10), and an interaction test for *q* exposures jointly, the exact set of *K* exposures, is performed (*q* = *K*). (**b**) One exposure is responsible for the full interaction effect (*K* = 1), and *q* is varied (*q* = 1, 2, 5 and 10). (**c**) Total interaction effect is partitioned equally among 10 exposures (*K* = 10), and *q* is varied within subsets of these 10 exposures (*q* = 1, 2, 5 and 10). (**d**) As in (a), with a single exposure simulated and tested, but varying the strength of the genetic main effect. GEI, gene–environment interaction; % V.E., percent variance explained

A power analysis was undertaken using a subset of independent variants from UKB chromosome 16. Using a subset of 100 variants and up to 10 random, continuous environmental factors, phenotypes were simulated to contain pre-specified signals reflecting genetic main effects, environmental main effects and GEI effects (see Methods; Supplementary Table S1). Statistical power from the simulations is shown in Figure 1. Power to detect single-exposure interaction effects was just over 70% for an overall $R_{G\times E,\;\mathrm{total}}^{2}$ of 1% (corresponding to a per-variant $R_{G\times E}^{2}$ of 0.01%), consistent with expectation based on theoretical power calculations. Power was reduced somewhat when the interaction test degrees of freedom increased, by either spreading the interaction effect across multiple exposures (Fig. 1a) or conducting multi-exposure interaction tests with only one true active environment (Fig. 1b). We note that these two simulations result in the same total variance explained by interaction effects from the generative models, with the same number of degrees of freedom in the tests, and thus have highly similar results. Power was decreased in the setting where true interaction signal was spread over 10 exposures but only a subset of these were included in multi-exposure interaction tests (Fig. 1c). A minor reduction in power was observed when variants contained a main-effect signal (per-variant $R_{G}^{2}$ of 0.1%; Fig. 1d).

### 3.2 Benchmarking for resource usage

**Fig. 2.**
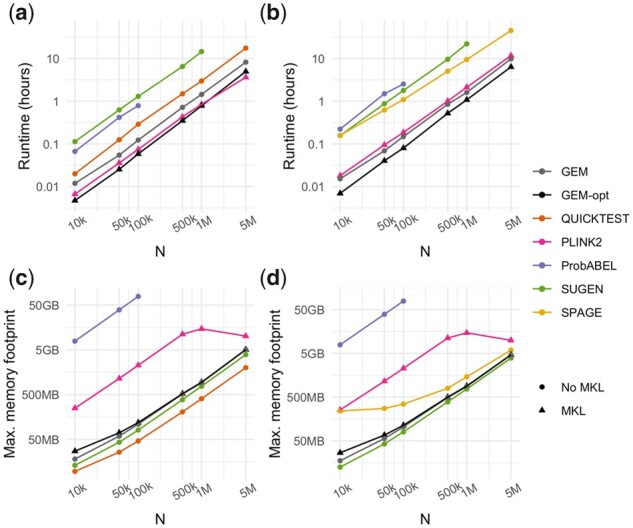
Benchmarking of GEM and other tools for GEI. Runtime (**a, b**) and maximum memory footprint (**c, d**) are shown as a function of sample size (*N*) and interaction testing program, using 100 000 simulated variants with the number of covariates held constant at three. The single exposure and outcome for each run were randomly simulated, with the outcome being either continuous (a, c) or binary with a case-control ratio of 1:3 (b, d). Circles and triangles correspond to programs compiled without or with Intel MKL, respectively. ‘GEM-opt’ refers to GEM runs using optimal parameters for speed, including compilation with MKL and pgen file inputs. All programs were run using a single thread and without robust standard errors. Results for ProbABEL at *N* > 100k were excluded because memory usage exceeded 100 GB

We compared run times and memory usage of the GEM software tool in comparison to the other software programs when adjusting for 3 covariates and using model-based (non-robust) standard errors (Fig. 2**)**. We also show the results of equivalent runs in terms of run time (Supplementary Fig. S4) and memory (Supplementary Fig. S5) using different numbers of covariates and robust standard errors. GEM run times were substantially faster than those of QUICKTEST, ProbABEL, SUGEN and SPAGE. GEM runtimes were similar to those of PLINK2 for continuous outcomes and somewhat faster for binary outcomes, and this advantage increased with the number of covariates (Supplementary Fig. S4). Though GEM and SPAGE both implement a matrix projection algorithm, GEM may be substantially faster due to its native implementation in C++. The low runtimes for PLINK2, despite implementing the classical model, are likely also due to a highly efficient C++ implementation rather than differences in the mathematical model. This interpretation is supported by the increasing advantage of both GEM and SPAGE compared to PLINK2 when many covariates are used (Supplementary Fig. S4). Memory usage was modest for all programs other than ProbABEL, whose memory requirements we found to be >100 GB for sample sizes greater than 100 000 in this analysis setup. Use of robust standard errors (where possible) did not change resource usages considerably other than for QUICKTEST, where run times approximately doubled (Supplementary Fig. S4). We further used a subset of this benchmarking dataset to compare *P*-values calculated using GEM versus ProbABEL, finding very high concordance for a random subset of 5000 variants (Supplementary Fig. S6).

### 3.3 Application to the GEI study for sex dimorphic genetic effects of WHR in UK Biobank

**Fig. 3.**
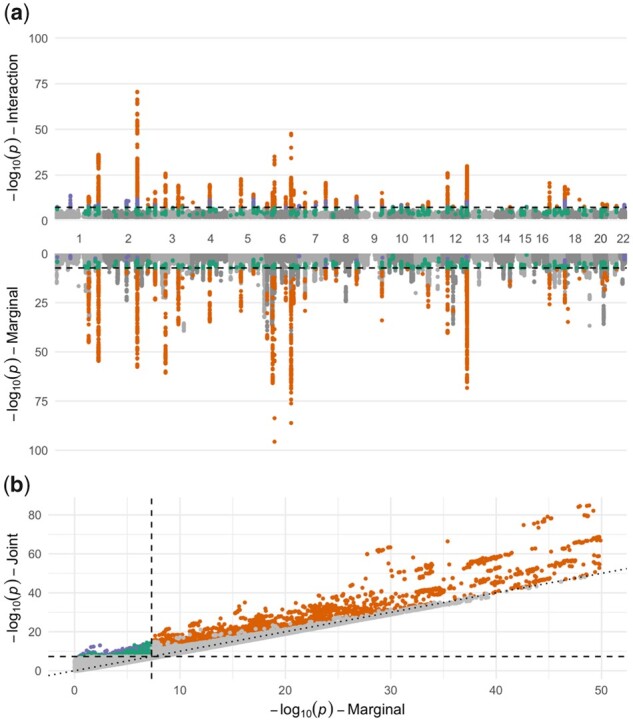
Results from genome-wide interaction analysis of WHR in the UK Biobank. (**a**) Two-sided Manhattan plot displays association strengths for the interaction test (here, G×sex interaction; top) and the marginal genetic effect test (from a model with no interaction; bottom). *x*-axis represents genomic position and *y*-axis represents the negative logarithm of the *P*-value for association at that locus. (**b**) Comparison of marginal and joint association strengths. The *x*-axis and *y*-axis show the negative logarithm of the association *P*-value using the marginal test (with no interaction) and the joint test, respectively. Dotted line corresponds to *y *=* x*. For both panels, dashed lines denote genome-wide significance thresholds (*P* < 5 × 10^−8^). Variants shown in orange passed a genome-wide significance threshold for both interaction and marginal effects. Variants shown in purple passed a genome-wide significance threshold for interaction effect, but not the marginal effect. Variants shown in green passed a genome-wide significance threshold using the joint test, but not for interaction nor marginal effects. For visualization purposes, variants with *P* < 1 × 10^−100^ were excluded from the Manhattan plot (from a single locus on chromosome 6 only), and variants with *P* < 1 × 10^−50^ were excluded from the joint plot

A genome-wide scan was then performed to test G×sex interactions influencing WHR in the UKB (Fig. 3, QQ plots in Supplementary Fig. S7). At a genome-wide significance threshold of 5×10^−8^, 257 independent loci were significant using the joint test (Supplementary Table S1; Supplementary Fig. S8). Of these, 29 did not have significant effects in the marginal test and 24 were not previously reported in the recently published BMI-adjusted WHR GWAS from the GIANT Consortium (Pulit *et al.*, 2019). This included loci at which neither the interaction effect test nor the marginal test were significant, such as that on chromosome 4 with the index variant rs10025536 (joint test *P* = 2.4×10^−9^), in an intron of *PDE5A*. When applying a more stringent multiple-testing threshold as in the meta-analysis (*P* < 5×10^−9^), 11 of these novel signals remained. Many variants showed a much lower association *P*-value from the joint test compared to that from the marginal test of genetic effect (Fig. 3b), though for 63% of the variants, the *P*-value for marginal association was lower than that for the joint test, as expected under the null hypothesis of no interaction.

In addition, 54 independent loci were uncovered using the G×sex interaction effect test (Supplementary Table S2). Of these, 9 were not detected with the marginal test (marginal *P*-values ranged from 2.7×10^−4^ to 0.25), and 6 were not previously reported as sex dimorphic in Pulit *et al.* (2019). Sensitivity analysis at these loci demonstrated excellent concordance with alternative software programs (Supplementary Fig. S9). The lowest interaction *P*-value was found on chromosome 2 at rs13389219 (*P* = 2.6×10^−71^), near the *SLC38A11* gene. As shown in Supplementary Figure S10 and Supplementary Table S4, the effect of this variant is more pronounced in females than males.

We also evaluated the impact of the interaction covariate (G×BMI term) on the genetic architecture of the G×sex interaction effect. We present a comparison between the *P*-values of the G×sex interaction test with and without the covariate in Supplementary Figure S11. Interaction covariate adjustment did not notably affect interaction strengths for these variants, though it did modestly strengthen the significance for interactions that were uniquely discovered in this analysis. To explore the impact of sample size on the genomic inflation observed here and assess polygenicity of interaction effects, the WHR analysis was re-run on three random down-sampled UK Biobank datasets (approximately 25% the size of the full sample; see Methods). Genomic inflation lambda values for marginal genetic effects in these down-sampled datasets were approximately equal to that of the GEI effects in the full sample (Supplementary Fig. S12).

Motivated by the investigation conducted by Winkler et al. (2018), which analyzed WHR interactions with both sex and age in stratified approach, we conducted an additional genome-wide scan to jointly test genetic interactions with sex and age (dichotomized as greater or less than 50 years old). We did not identify any additional loci using this approach, finding 41 significant loci after pruning (a strict subset of the 54 that were identified when using sex alone as an interaction). This observation illustrates the impact of using additional degrees of freedom in multiple-exposure interaction testing in the absence of substantial expected interaction effects. Thus, while the importance of simultaneous multi-exposure testing in some settings has been described previously (Kim *et al.*, 2019), users should take this statistical power cost into consideration when designing interaction analyses.

## 4 Discussion

GEI testing is becoming increasingly important for improving biological understanding and enabling precision medicine, but current tools do not typically enable an optimal set of statistical methods while retaining scalability to millions of samples. To approach this problem, we have introduced GEM, a software program intended for large-scale, genome-wide GEI analysis using a single variant testing approach. We showed that GEM maintains reasonable power and type I error under a variety of simulation settings, and that GEM performs well compared to existing programs for GEI studies in terms of both runtime and memory usage while including important statistical capabilities. We further used GEM to conduct a genome-wide analysis of G×sex interaction influencing WHR in the UK Biobank, and found that the inclusion of explicit interactions in the statistical model enhanced discovery in comparison to a stratified approach.

One major advantage of GEM is its ability to incorporate multiple interaction terms. This enables proper control for genotype-covariate effects (Keller, 2014) as well as multi-exposure interaction tests. These are conducted as *q*-degrees of freedom Chi-square tests, where *q* is the number of environmental exposures whose interaction terms are to be included. Interaction analysis incorporating contributions from multiple exposures has been described previously, using both fixed effects (‘omnibus’ test) (Kim *et al.*, 2019) and random effects (flexibly summarizing the contributions of multiple environments as a similarity matrix) (Moore *et al.*, 2019) approaches. Each of these approaches show an increase in statistical power when the underlying model indeed included multiple active environments, an observation which was replicated in the current study with the multi-exposure test implemented in GEM. We further demonstrated that power loss was modest when performing multi-exposure tests with only one true active environment interaction (Fig. 1b). This suggests that the inclusion of multiple exposures in interaction studies with very large sample sizes may be a promising approach to discover additional loci and expand the genetic architecture of complex diseases.

In benchmarking tests using simulated data, GEM demonstrated the feasibility of genome-wide GEI scans in large-scale cohorts of up to millions of samples. For example, it took less than one hour to conduct a robust analysis of a continuous phenotype for 100 000 variants in 1 million samples using a single thread, while requiring approximately 1 GB of memory. Based on the benchmarking results and current program specifications (Table 1), we found that GEM is the only option that permits efficient GEI analyses in millions of samples while allowing for robust standard errors and the inclusion of multiple interaction terms. We also note that the run times of GEM and PLINK2 can be improved considerably through the use of multithreading when multiple computing cores are available.

We used anthropometric traits from the UK Biobank to conduct a genome-wide interaction analysis with GEM and evaluated G×sex interactions influencing WHR. We report 54 independent loci supporting interactions with sex, with the strongest near the *SLC38A11* gene which has been previously linked to impedance-based body fat distribution in the UK Biobank (Rask-Andersen *et al.*, 2019). Interaction analyses have been performed in the UK Biobank for anthropometric traits (primarily BMI), but have generally used polygenic marginal-effect scores as the genetic effect, possibly due to the computational limitations described here (Rask-Andersen *et al.*, 2017; Calvin *et al.*, 2019; Tyrrell et al., 2017). This polygenic score-based approach may improve power when marginal and interaction effects are correlated, but otherwise may impair discovery (Kim *et al.*, 2019). The largest GWAS meta-analysis of BMI-adjusted WHR to date includes the UK Biobank to reach a total sample size of 694 649 and reports many genetic effect differences between males and females using a stratified approach (Pulit *et al.*, 2019). While sex-dimorphism of genetic effects has been reported for many traits, such as type 2 diabetes (Morris *et al.*, 2012) and longevity (Zeng *et al.*, 2018), such effects are stronger and more numerous for WHR.

We demonstrate the value of the GEM method by detecting 6 additional loci with the interaction test among 352 768 participants from the UK Biobank study and detecting an additional 24 loci with the joint test that did not pass the genome-wide significance threshold in either the combined or stratified main-effect analyses from Pulit *et al.* (2019). This increased discovery could be due to the fact that the joint approach implemented in GEM is equivalent to testing the hypothesis: $\beta_{\mathrm{male}}=\beta_{\mathrm{female}}=0$, which is more powerful in the presence of G×sex interaction than tests of the of the combined ($\beta_{\mathrm{combined}}=0)$ or stratified ($\beta_{\mathrm{male}}=0$ and $\beta_{\mathrm{female}}=0$ separately) effects. This added value is particularly notable, given that the present analysis was conducted in a subset of individuals (UKB) contributing only approximately half of the total meta-analysis sample size. While adjustment for interaction covariates (such as the G×BMI term included here) may theoretically enhance discovery by reducing residual noise or reduce false positive findings due to confounding, we did not observe a major influence of this adjustment in our analysis. In addition, our analysis of genomic inflation in down-sampled UK Biobank subsets provides preliminary empirical evidence that the genetic architecture of marginal and GEI effects is similar, while confirming prior findings that approximately four times the sample size may be required to detect GEI effects with the same power as an equivalent marginal analysis.

In its current form, GEM does not account for relatedness, so it may produce biased estimates unless the population is filtered for unrelated individuals. Another limitation is the known invalidity of asymptotic tests from logistic models for binary traits in the presence of substantial case-control imbalance. The authors of SPAGE found that for common variants, type I error rates under the same basic matrix projection model deteriorate with case-control ratios less than roughly 1:9 (Bi *et al.*, 2019). For more extreme case-control imbalance, we suggest the use of SPAGE, which uses a saddlepoint approximation to calibrate score test statistics.

In summary, we have described a new software program, GEM, for conducting genome-wide GEI testing in datasets of up to millions of individuals, while allowing for multiple exposures and robust standard errors. We have made the software (https://github.com/large-scale-gxe-methods/GEM) and workflows (https://github.com/large-scale-gxe-methods/gem-workflow) available in publicly available repositories. GEM facilitates the next generation of large-scale and consortium-based GEI studies and thus enables important discoveries for genomic understanding and personalized medicine.

## Acknowledgements

This research was conducted using the UK Biobank Resource under Application Numbers 27892 and 42646. K.E.W. and A.K.M. completed the analysis of UK Biobank data independently of their capacity as employees of Mass General Brigham. Some simulation studies were performed in the Analysis Commons on DNAnexus, a hosting platform that uses Amazon Web Services (AWS) to provide a cloud data management and computing environment for large genomic data projects.

### Author Contributions

D.T.P., L.H., H.C. and A.K.M. developed the GEM algorithm. D.T.P., L.H. and H.C. implemented the GEM software program. K.E.W., D.T.P., Y.C., M.S.-G and A.K.M. implemented software programs as cloud workflows. K.E.W., H.C. and A.K.M. designed the simulations and experiments. K.E.W. and D.T.P. carried out the simulations and data analyses. Y.J.S., Y.V.S. and A.C.M. provided guidance on experimental design and data analyses. K.E.W., H.C. and A.K.M. wrote the manuscript. All authors critically read the manuscript.

## Funding

This work was supported by National Institutes of Health (NIH) [grant number R01 HL145025]. The Analysis Commons was funded by NIH [grant number R01 HL131136].

*Conflict of Interest*: none declared.

## Supplementary Material

btab223_Supplementary_Data
