# Supplementary material for: GEM: scalable and flexible gene–environment interaction analysis in millions of samples
Source: Bioinformatics. 2021 May 25;37(20):3514–20. doi: 10.1093/bioinformatics/btab223 (PMC8545347; doi:10.1093/bioinformatics/btab223)
Supplement: btab223_Supplementary_Data [file btab223_supplementary_data.pdf]

GEM: Scalable and Flexible Gene-Environment Interaction Analysis in Millions of Samples

Kenneth E. Westerman, Duy T. Pham, Liang Hong, Ye Chen, Magdalena Sevilla-Gonzalez, Yun Ju Sung, Yan V. Sun, Alanna C. Morrison, Han Chen, Alisa K. Manning

1 Supplementary Methods

Consider a generalized linear model for N unrelated individuals

$$g(\mu_i) = \boldsymbol{X}_i\boldsymbol{\beta}_X + G_i\beta_G + \boldsymbol{C}_i\boldsymbol{\beta}_C + \boldsymbol{S}_i\boldsymbol{\beta}_S, \tag{1}$$

where $\mu_i = E(Y_i|\boldsymbol{X}_i, G_i)$ is the conditional mean of the phenotype Y_i for individual i given covariates $\boldsymbol{X}_i$ (including an intercept for the model) and the genotype G_i of a single genetic variant. The gene-environment interaction terms $\boldsymbol{C}_i$ and $\boldsymbol{S}_i$ are the products of G_i and c and q environmental terms (which are included in the covariates $\boldsymbol{X}_i$), respectively. The link function $g(\cdot)$ is a monotone function (usually the identity link function for continuous phenotypes, and the logit link function for binary phenotypes).

Let $\boldsymbol{Y} = (Y_1 \quad Y_2 \quad \dots \quad Y_N)^T$ be a length N vector of the phenotypes, $\boldsymbol{X} = (\boldsymbol{X}_1^T \quad \boldsymbol{X}_2^T \quad \dots \quad \boldsymbol{X}_N^T)^T$ be an $N \times p$ matrix of p covariates (including an intercept for the model and all the $c+q$ environmental terms that interact with the genotype), $\boldsymbol{G} = (G_1 \quad G_2 \quad \dots \quad G_N)^T$ be a length N vector of the genotype for this single genetic variant, $\boldsymbol{C} = (\boldsymbol{C}_1^T \quad \boldsymbol{C}_2^T \quad \dots \quad \boldsymbol{C}_N^T)^T$ be an $N \times c$ matrix of c gene-environment interaction terms that are adjusted for as covariates, $\boldsymbol{S} = (\boldsymbol{S}_1^T \quad \boldsymbol{S}_2^T \quad \dots \quad \boldsymbol{S}_N^T)^T$ be an $N \times q$ matrix of q gene-environment interaction terms of interest in the gene-environment interaction test ($H_0 : \boldsymbol{\beta}_S = \boldsymbol{0}$ versus $H_1 : \boldsymbol{\beta}_S \neq \boldsymbol{0}$), we fit a null model without any genetic effects

$$g(\mu_i) = \boldsymbol{X}_i\boldsymbol{\beta}_X. \tag{2}$$

For a linear regression model, we can get the estimate $\hat{\boldsymbol{\beta}}_X = (\boldsymbol{X}^T\boldsymbol{X})^{-1}\boldsymbol{X}^T\boldsymbol{Y}$ and the length N residual vector $\boldsymbol{r} = \boldsymbol{Y} - \boldsymbol{X}\hat{\boldsymbol{\beta}}_X$, as well as the residual variance estimate $\hat{\sigma}^2 = \frac{\boldsymbol{r}^T\boldsymbol{r}}{N-p}$. For a logistic regression model, we can iteratively solve the problem until convergence, with the estimate $\hat{\boldsymbol{\beta}}_X = (\boldsymbol{X}^T\boldsymbol{W}\boldsymbol{X})^{-1}\boldsymbol{X}^T\boldsymbol{W}\tilde{\boldsymbol{Y}}$, where $\boldsymbol{W}$ is a diagonal matrix with elements $\hat{\mu}_i(1-\hat{\mu}_i)$, with $\hat{\mu}_i = \frac{exp(\boldsymbol{X}_i\hat{\boldsymbol{\beta}}_X)}{1+exp(\boldsymbol{X}_i\hat{\boldsymbol{\beta}}_X)}$ being the estimate of $P(Y_i = 1|\boldsymbol{X}_i, G_i)$ for individual i, and also the length N residual vector $\boldsymbol{r} = \boldsymbol{Y} - \hat{\boldsymbol{\mu}}$, where $\hat{\boldsymbol{\mu}} = (\hat{\mu}_1 \quad \hat{\mu}_2 \quad \dots \quad \hat{\mu}_N)^T$ is a length N

vector of these estimated phenotype (disease) probabilities, $\tilde{\boldsymbol{Y}} = \boldsymbol{X}\hat{\boldsymbol{\beta}}_X + \boldsymbol{W}^{-1}\boldsymbol{r}$ is the working vector at convergence.

For each genetic variant $\boldsymbol{G}$ in a linear regression model, we first compute $\tilde{\boldsymbol{G}} = \boldsymbol{G} - \boldsymbol{X}(\boldsymbol{X}^T\boldsymbol{X})^{-1}\boldsymbol{X}^T\boldsymbol{G}$, $\tilde{\boldsymbol{C}} = \boldsymbol{C} - \boldsymbol{X}(\boldsymbol{X}^T\boldsymbol{X})^{-1}\boldsymbol{X}^T\boldsymbol{C}$ and $\tilde{\boldsymbol{S}} = \boldsymbol{S} - \boldsymbol{X}(\boldsymbol{X}^T\boldsymbol{X})^{-1}\boldsymbol{X}^T\boldsymbol{S}$, and estimate the marginal genetic effect $\tilde{\beta}_G = (\tilde{\boldsymbol{G}}^T\tilde{\boldsymbol{G}})^{-1}\tilde{\boldsymbol{G}}^T\boldsymbol{r}$ with a model-based variance estimate $Var(\tilde{\beta}_G) = \hat{\sigma}^2(\tilde{\boldsymbol{G}}^T\tilde{\boldsymbol{G}})^{-1}$ or a robust (sandwich) variance estimate $Var_R(\tilde{\beta}_G) = (\tilde{\boldsymbol{G}}^T\tilde{\boldsymbol{G}})^{-1}\tilde{\boldsymbol{G}}^T\boldsymbol{D}\tilde{\boldsymbol{G}}(\tilde{\boldsymbol{G}}^T\tilde{\boldsymbol{G}})^{-1}$, where $\boldsymbol{D}$ is a diagonal matrix with elements r_i^2 (r_i is the ith element of the residual vector $\boldsymbol{r}$). Let $\boldsymbol{U} = (\tilde{\boldsymbol{G}} \quad \tilde{\boldsymbol{C}} \quad \tilde{\boldsymbol{S}})^T\boldsymbol{r}$ be a length $(1 + c + q)$ vector ($c \geq 0$), $\boldsymbol{V} = (\tilde{\boldsymbol{G}} \quad \tilde{\boldsymbol{C}} \quad \tilde{\boldsymbol{S}})^T(\tilde{\boldsymbol{G}} \quad \tilde{\boldsymbol{C}} \quad \tilde{\boldsymbol{S}})$ and $\boldsymbol{\Omega} = (\tilde{\boldsymbol{G}} \quad \tilde{\boldsymbol{C}} \quad \tilde{\boldsymbol{S}})^T\boldsymbol{D}(\tilde{\boldsymbol{G}} \quad \tilde{\boldsymbol{C}} \quad \tilde{\boldsymbol{S}})$ be $(1+c+q) \times (1+c+q)$ matrices, we then jointly estimate the genetic main effect and gene-environment interaction effects $\hat{\boldsymbol{\beta}}_{G,C,S} = (\hat{\beta}_G \quad \hat{\boldsymbol{\beta}}_C^T \quad \hat{\boldsymbol{\beta}}_S^T)^T = \boldsymbol{V}^{-1}\boldsymbol{U}$, with a model-based covariance matrix estimate $Cov(\hat{\boldsymbol{\beta}}_{G,C,S}) = \hat{\sigma}^2\boldsymbol{V}^{-1}$ or a robust (sandwich) covariance matrix estimate $Cov_R(\hat{\boldsymbol{\beta}}_{G,C,S}) = \boldsymbol{V}^{-1}\boldsymbol{\Omega}\boldsymbol{V}^{-1}$.

Similarly, for a logistic regression model, we first compute the quantities $\tilde{\boldsymbol{G}} = \boldsymbol{G} - \boldsymbol{X}(\boldsymbol{X}^T\boldsymbol{W}\boldsymbol{X})^{-1}\boldsymbol{X}^T\boldsymbol{W}\boldsymbol{G}$, $\tilde{\boldsymbol{C}} = \boldsymbol{C} - \boldsymbol{X}(\boldsymbol{X}^T\boldsymbol{W}\boldsymbol{X})^{-1}\boldsymbol{X}^T\boldsymbol{W}\boldsymbol{C}$ and $\tilde{\boldsymbol{S}} = \boldsymbol{S} - \boldsymbol{X}(\boldsymbol{X}^T\boldsymbol{W}\boldsymbol{X})^{-1}\boldsymbol{X}^T\boldsymbol{W}\boldsymbol{S}$, and estimate the marginal genetic effect $\tilde{\beta}_G = (\tilde{\boldsymbol{G}}^T\boldsymbol{W}\tilde{\boldsymbol{G}})^{-1}\tilde{\boldsymbol{G}}^T\boldsymbol{r}$ with a model-based variance estimate $Var(\tilde{\beta}_G) = (\tilde{\boldsymbol{G}}^T\boldsymbol{W}\tilde{\boldsymbol{G}})^{-1}$ or a robust variance estimate $Var_R(\tilde{\beta}_G) = (\tilde{\boldsymbol{G}}^T\boldsymbol{W}\tilde{\boldsymbol{G}})^{-1}\tilde{\boldsymbol{G}}^T\boldsymbol{D}\tilde{\boldsymbol{G}}(\tilde{\boldsymbol{G}}^T\boldsymbol{W}\tilde{\boldsymbol{G}})^{-1}$, where $\boldsymbol{D}$ is a diagonal matrix with elements r_i^2 (r_i is the ith element of the residual vector $\boldsymbol{r}$). Let $\boldsymbol{U} = (\tilde{\boldsymbol{G}} \quad \tilde{\boldsymbol{C}} \quad \tilde{\boldsymbol{S}})^T\boldsymbol{r}$ be a length $(1+c+q)$ vector ($c \geq 0$), $\boldsymbol{V} = (\tilde{\boldsymbol{G}} \quad \tilde{\boldsymbol{C}} \quad \tilde{\boldsymbol{S}})^T\boldsymbol{W}(\tilde{\boldsymbol{G}} \quad \tilde{\boldsymbol{C}} \quad \tilde{\boldsymbol{S}})$ and $\boldsymbol{\Omega} = (\tilde{\boldsymbol{G}} \quad \tilde{\boldsymbol{C}} \quad \tilde{\boldsymbol{S}})^T\boldsymbol{D}(\tilde{\boldsymbol{G}} \quad \tilde{\boldsymbol{C}} \quad \tilde{\boldsymbol{S}})$ be $(1+c+q) \times (1+c+q)$ matrices, we then jointly estimate the genetic main effect and gene-environment interaction effects $\hat{\boldsymbol{\beta}}_{G,C,S} = (\hat{\beta}_G \quad \hat{\boldsymbol{\beta}}_C^T \quad \hat{\boldsymbol{\beta}}_S^T)^T = \boldsymbol{V}^{-1}\boldsymbol{U}$, with a model-based covariance matrix estimate $Cov(\hat{\boldsymbol{\beta}}_{G,C,S}) = \boldsymbol{V}^{-1}$ or a robust (sandwich) covariance matrix estimate $Cov_R(\hat{\boldsymbol{\beta}}_{G,C,S}) = \boldsymbol{V}^{-1}\boldsymbol{\Omega}\boldsymbol{V}^{-1}$.

As the sample size is very large, we compute asymptotic p-values for both linear and logistic regression models. Specifically, under the null hypothesis of no marginal genetic effects ($H_0 : \beta_G = 0$), the marginal genetic effect test statistic $\frac{\tilde{\beta}_G^2}{Var(\tilde{\beta}_G)}$ (or the robust version $\frac{\tilde{\beta}_G^2}{Var_R(\tilde{\beta}_G)}$) follows a χ^2 distribution with 1 degree of freedom. Let $Cov(\hat{\boldsymbol{\beta}}_S)$ and $Cov_R(\hat{\boldsymbol{\beta}}_S)$ be $q \times q$ submatrices of $Cov(\hat{\boldsymbol{\beta}}_{G,C,S})$ and $Cov_R(\hat{\boldsymbol{\beta}}_{G,C,S})$, respectively, corresponding to the q gene-environment interaction effect estimates of interest $\hat{\boldsymbol{\beta}}_S$, under the null hypothesis of no gene-environment interactions of interest ($H_0 : \boldsymbol{\beta}_S = \boldsymbol{0}$), the interaction test statistic $\hat{\boldsymbol{\beta}}_S^T Cov(\hat{\boldsymbol{\beta}}_S)^{-1}\hat{\boldsymbol{\beta}}_S$ (or the robust version $\hat{\boldsymbol{\beta}}_S^T Cov_R(\hat{\boldsymbol{\beta}}_S)^{-1}\hat{\boldsymbol{\beta}}_S$) follows a χ^2 distribution with q degrees of freedom. Define $\hat{\boldsymbol{\beta}}_{G,S} = (\hat{\beta}_G \quad \hat{\boldsymbol{\beta}}_S^T)^T$, and let $Cov(\hat{\boldsymbol{\beta}}_{G,S})$ and $Cov_R(\hat{\boldsymbol{\beta}}_{G,S})$ be $(1+q) \times (1+q)$ submatrices of $Cov(\hat{\boldsymbol{\beta}}_{G,C,S})$ and $Cov_R(\hat{\boldsymbol{\beta}}_{G,C,S})$, respectively, corresponding to $\hat{\boldsymbol{\beta}}_{G,S}$, under the null hypothesis of no genetic main effects or gene-environment interactions of interest ($H_0 : \beta_G = 0$ and $\boldsymbol{\beta}_S = \boldsymbol{0}$), the joint test statistic $\hat{\boldsymbol{\beta}}_{G,S}^T Cov(\hat{\boldsymbol{\beta}}_{G,S})^{-1}\hat{\boldsymbol{\beta}}_{G,S}$ (or the robust ver-

sion $\hat{\boldsymbol{\beta}}_{G,S}^T Cov_R(\hat{\boldsymbol{\beta}}_{G,S})^{-1}\hat{\boldsymbol{\beta}}_{G,S})$ follows a χ^2 distribution with $(1+q)$ degrees of freedom.

2 Supplementary Figures

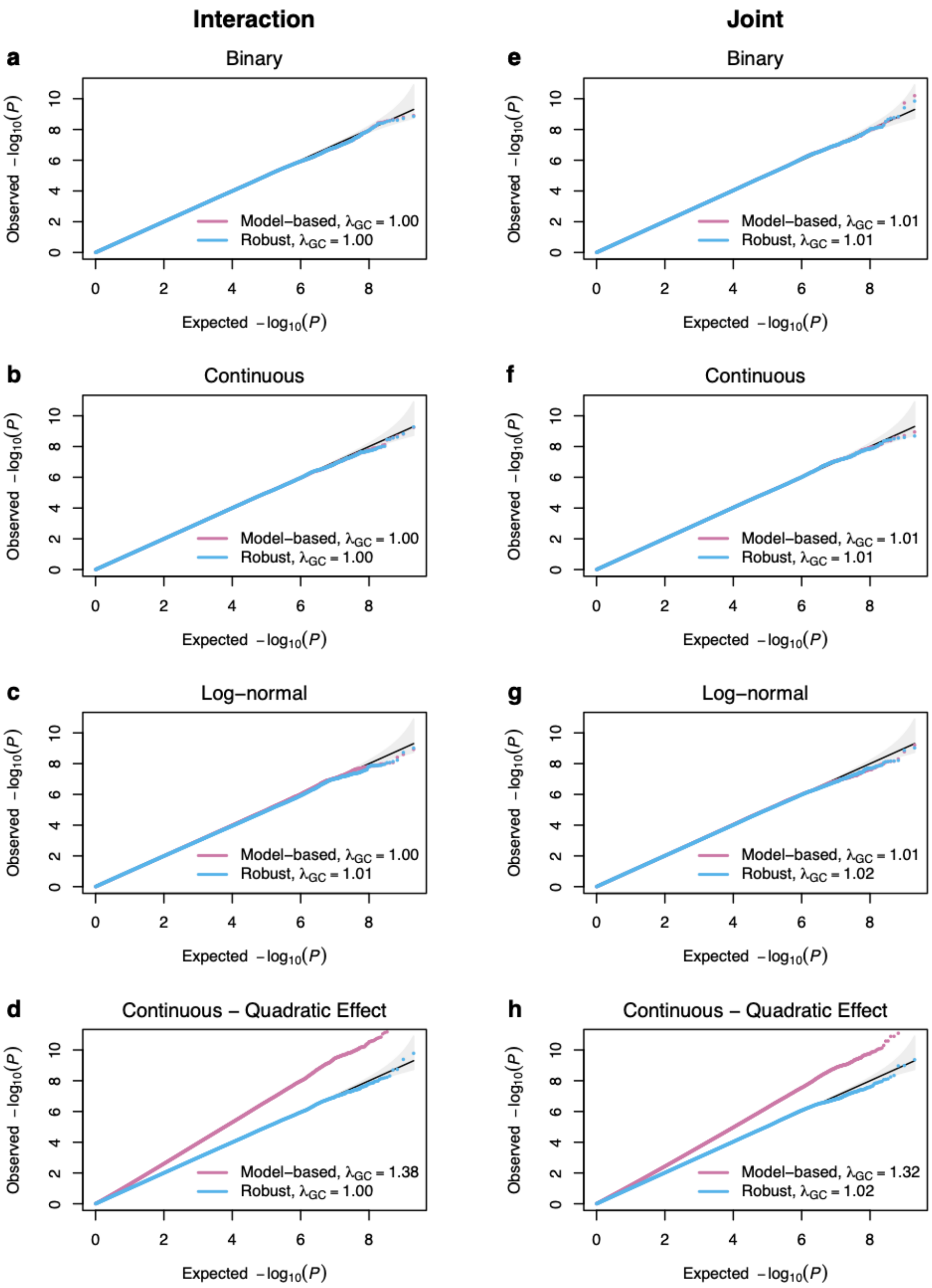

Supplementary Figure S1: Quantile-quantile plots from type I error simulations with no genetic effects. Plots compare observed versus expected p-value distributions for genetic variants on chromosomes 1-20, for which no genetic effects were simulated. Plots on the left correspond to the interaction test, and plots on the right correspond to the joint test. Plot titles denote the simulated distribution for the exposure, and panels (d) and (h) are based on phenotypes containing a mis-specified environmental main effect. Colors correspond to the approach used to calculate standard errors (model-based vs. robust).

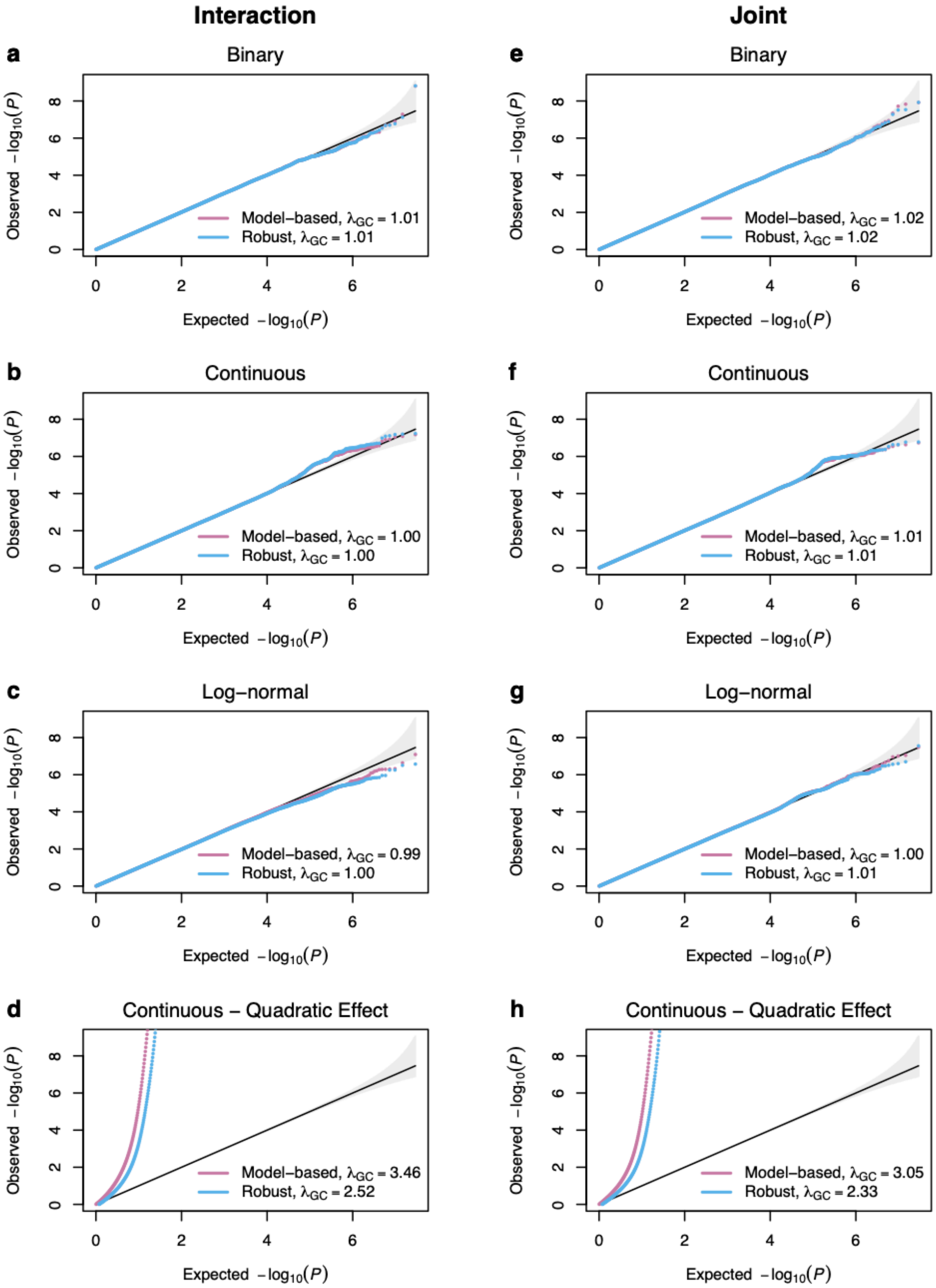

Supplementary Figure S2: Quantile-quantile plots from type I error simulations with gene-environment correlation. Plots compare observed versus expected p-value distributions for genetic variants on chromosome 21, for which 100 random variants explained a total of 10% of the exposure variance. Plots on the left correspond to the interaction test, and plots on the right correspond to the joint test. Plot titles denote the simulated distribution for the exposure, and panels (d) and (h) are based on phenotypes containing a mis-specified environmental main effect. Colors correspond to the approach used to calculate standard errors (model-based vs. robust).

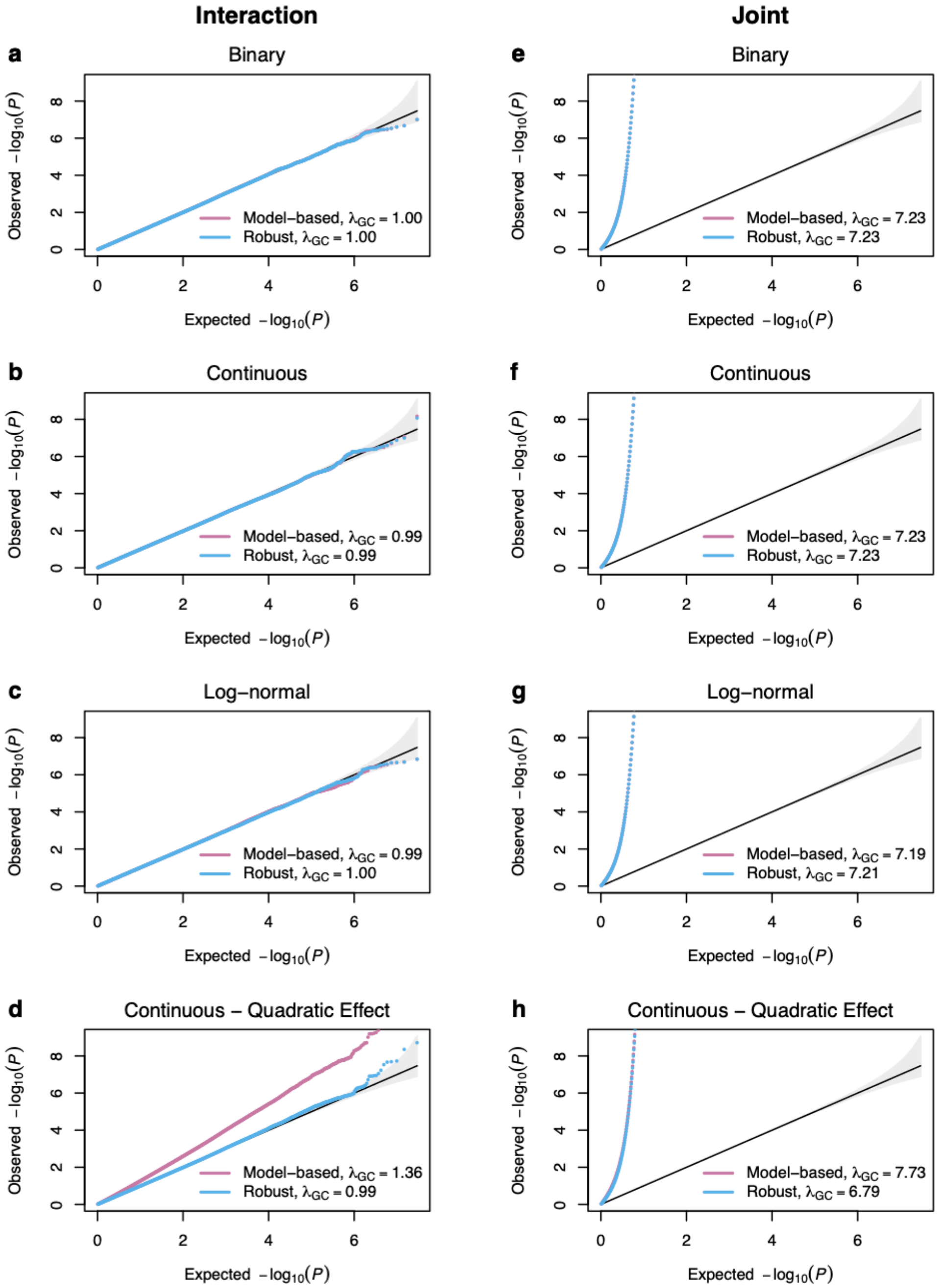

Supplementary Figure S3: Quantile-quantile plots from type I error simulations with genetic main effects. Plots compare observed versus expected p-value distributions for genetic variants on chromosome 22, for which 100 random variants explained a total of 10% of the phenotypic variance. Plots on the left correspond to the interaction test, and plots on the right correspond to the joint test. Plot titles denote the simulated distribution for the exposure, and panels (d) and (h) are based on phenotypes containing a mis-specified environmental main effect. Colors correspond to the approach used to calculate standard errors (model-based vs. robust).

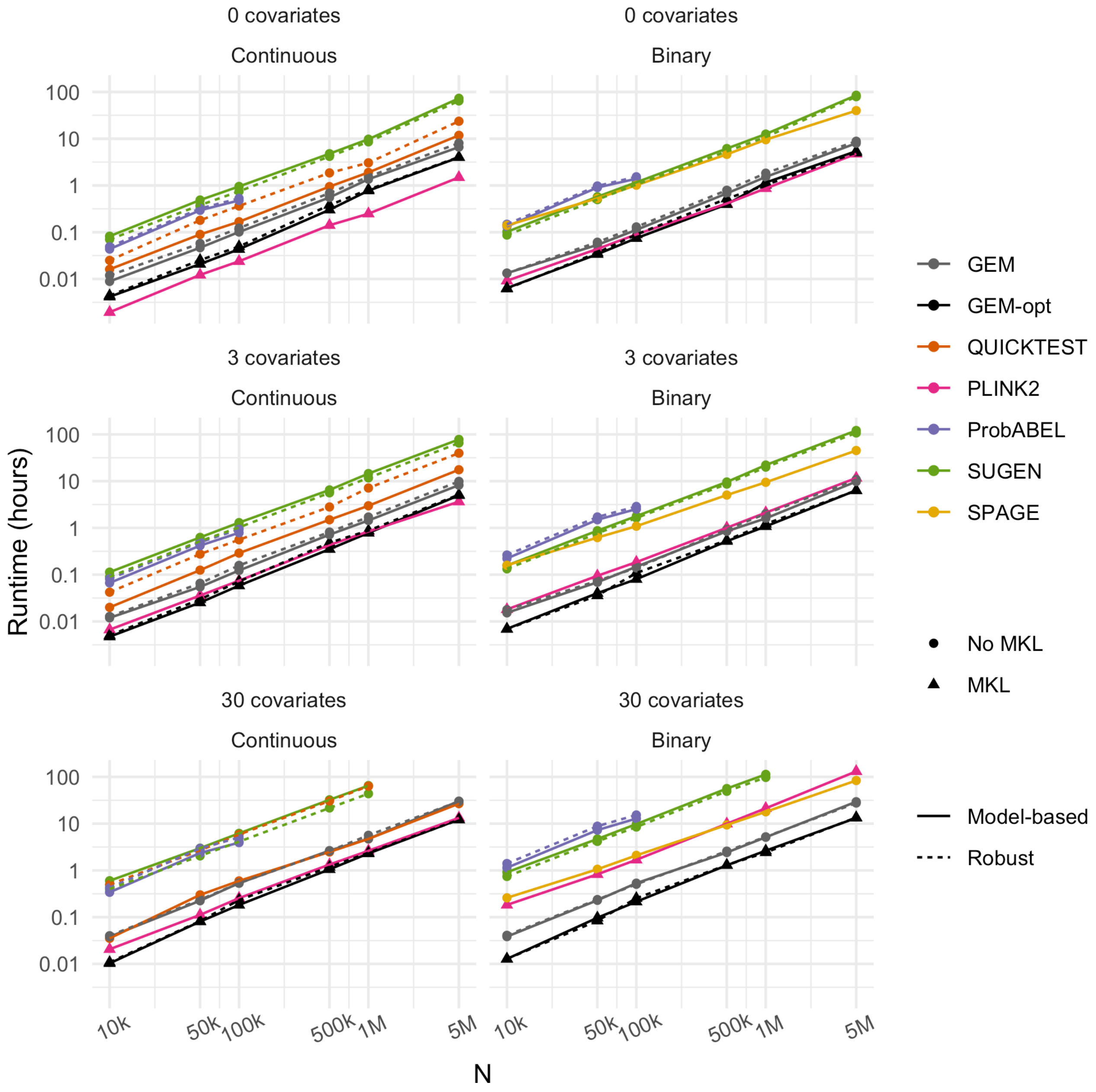

Supplementary Figure S4: Benchmarking of GEM and alternative software programs for run time with a continuous or binary outcome and varying number of covariates using 100,000 simulated variants. Run time is shown as a function of sample size (x-axis). Numbers above each panel correspond to the number of non-exposure covariates (top) and outcome type (bottom). Colors correspond to software program and dashed lines correspond to runs that used the option to obtain robust standard errors. Circles and triangles correspond to programs compiled without or with Intel MKL, respectively. "GEM-opt" denotes GEM runs using optimal parameters for speed, including compilation with MKL and pgen file inputs. Data points are not shown for runs that exceeded 100GB of memory (ProbABEL, N>100k), or were unfinished after 7 days (SUGEN, N=5M, 30 covariates; QUICKTEST, N=5M, 30 covariates, robust).

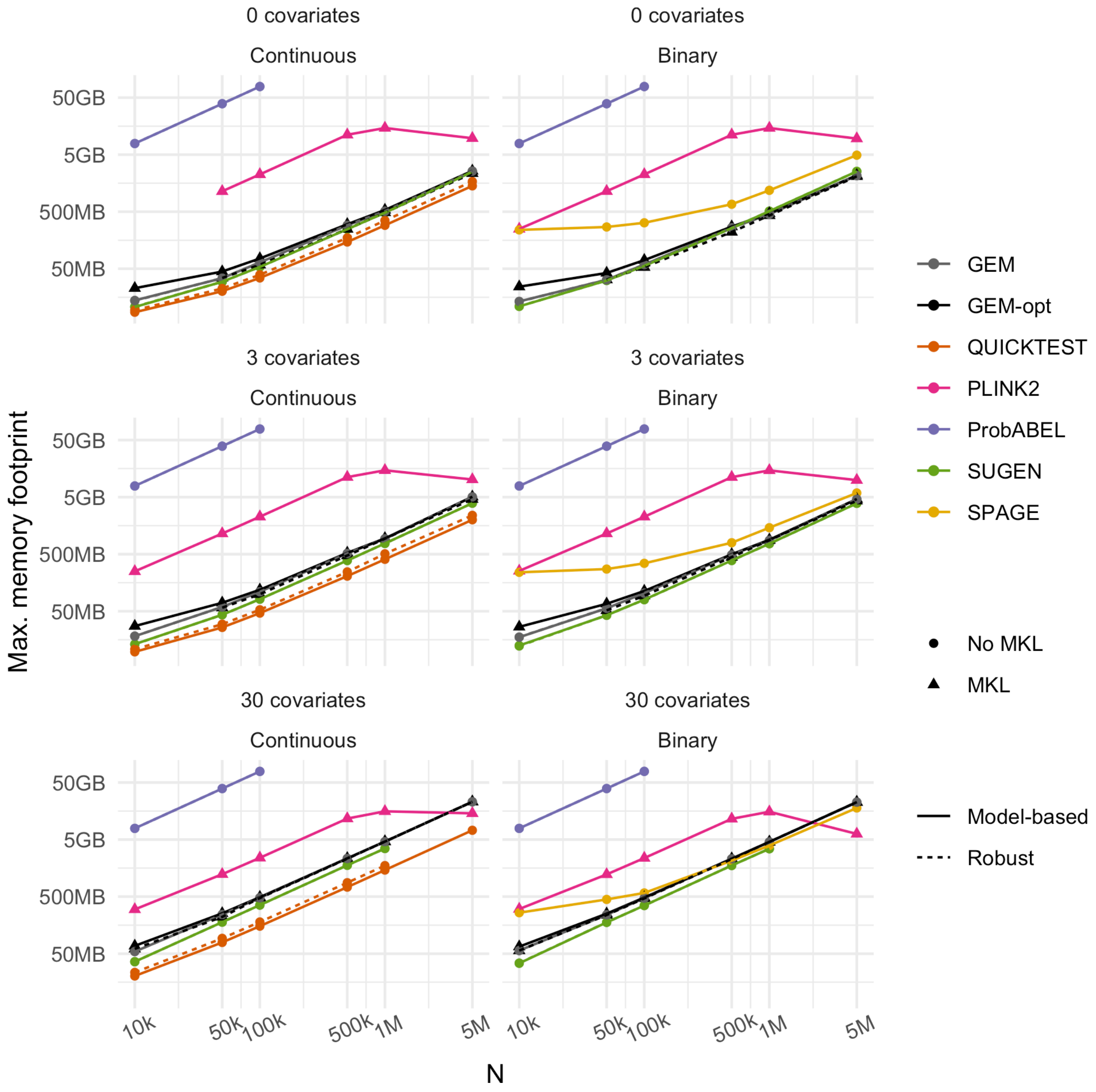

Supplementary Figure S5: Benchmarking of GEM and alternative software programs for memory usage with a continuous or binary outcome and varying number of covariates using 100,000 simulated variants. Maximum memory footprint is shown as a function of sample size (x-axis). Numbers above each panel correspond to the number of non-exposure covariates (top) and outcome type (bottom). Colors correspond to software program and dashed lines correspond to runs that used the option to obtain robust standard errors. Circles and triangles correspond to programs compiled without or with Intel MKL, respectively. "GEM-opt" denotes GEM runs using optimal parameters for speed, including compilation with MKL and pgen file inputs. Data points are not shown for runs that exceeded 100GB of memory (ProbABEL, N>100k), were unfinished after 7 days (SUGEN, N=5M, 30 covariates; QUICKTEST, N=5M, 30 covariates, robust), or some that took less than 10 seconds to run (PLINK2, N=10k, continuous, 0 covariates; GEM-opt, N=10k, 0 covariates).

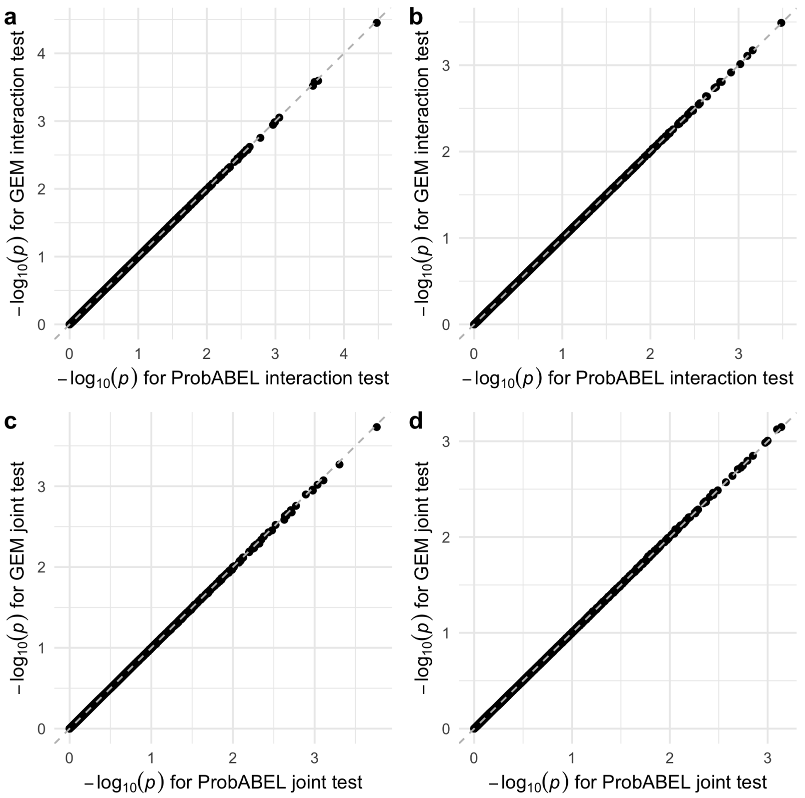

Supplementary Figure S6: Concordance of GEM and ProbABEL results. Regression coefficients were retrieved for a random 5000 variants from benchmarking runs using 3 covariates and a sample size of 10,000. Y- and x-axes display log p-values from GEM and ProbABEL, respectively. Panels correspond to the interaction test with continuous outcome (a), interaction test with binary outcome (b), joint test with continuous outcome (c), and joint test with binary outcome (d). Gray dashed line represents y = x.

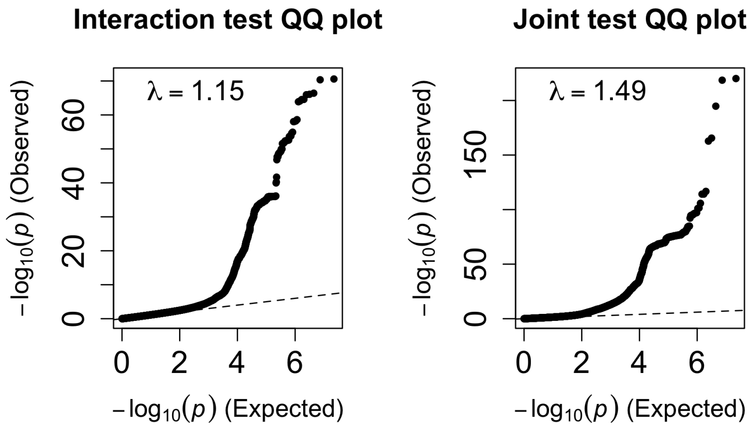

Supplementary Figure S7: Quantile-quantile p-value plots from the UK Biobank waist-hip ratio analysis. Panels correspond to the gene-environment interaction test (a) and joint test (b). Y-axis and x-axis display negative logarithms of association p-values that were observed and expected (based on a uniform distribution), respectively. Lambda values correspond to genomic inflation statistics (median chi-squared statistic / chi-squared statistic corresponding to p = 0.5).

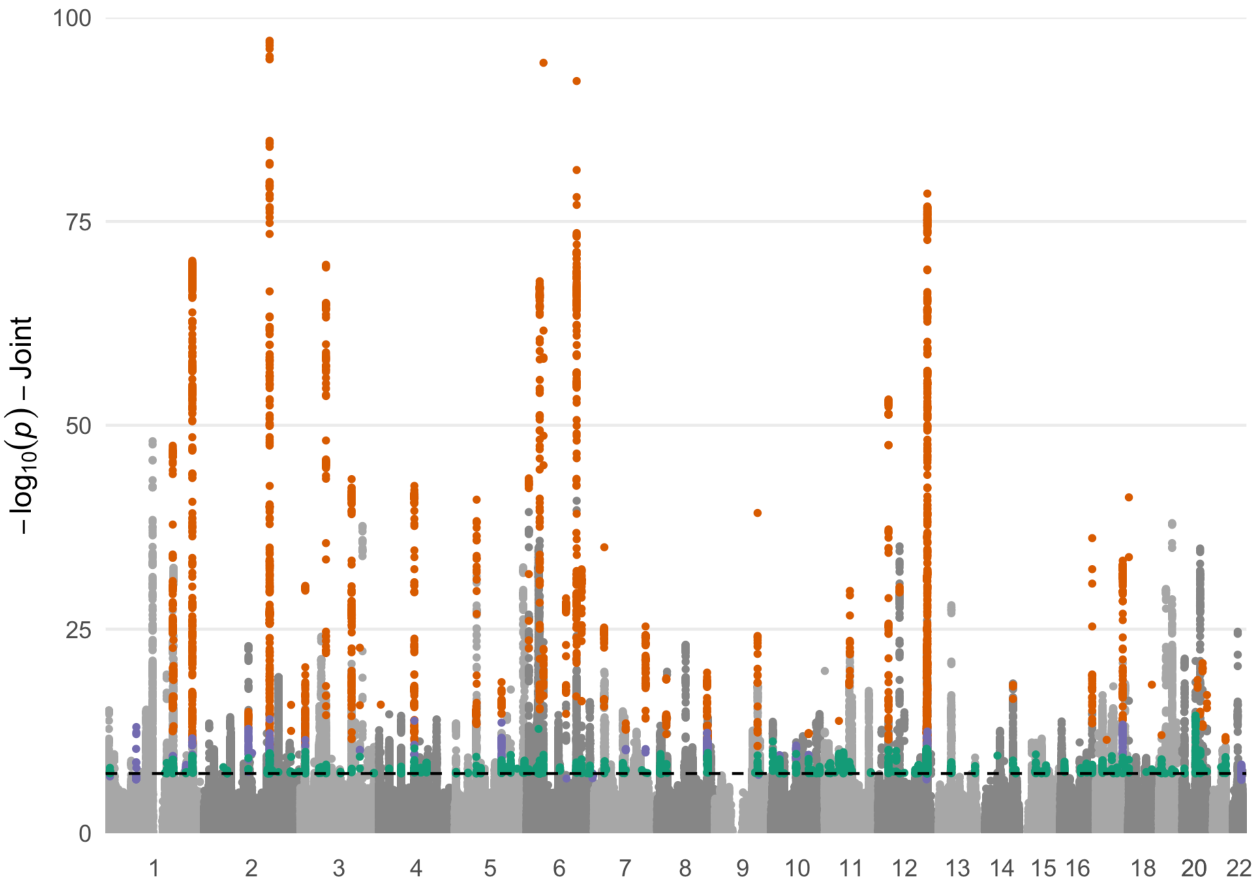

Supplementary Figure S8: Manhattan plot displays association strengths for the joint test of genetic main effect and $G \times sex$ interaction effect. x-axis represents genomic position and y-axis represents the negative logarithm of the p-value for association at that locus. Dashed line denotes the genome-wide significance threshold ($p < 5\times10^{-8}$). Variants shown in orange passed a genome-wide significance threshold for both interaction and marginal effects. Variants shown in purple passed a genome-wide significance threshold for interaction effect, but not the marginal effect. Variants shown in green passed a genome-wide significance threshold using the joint test, but not for interaction nor marginal effects. For visualization purposes, variants with $p < 1\times10^{-100}$ were excluded.

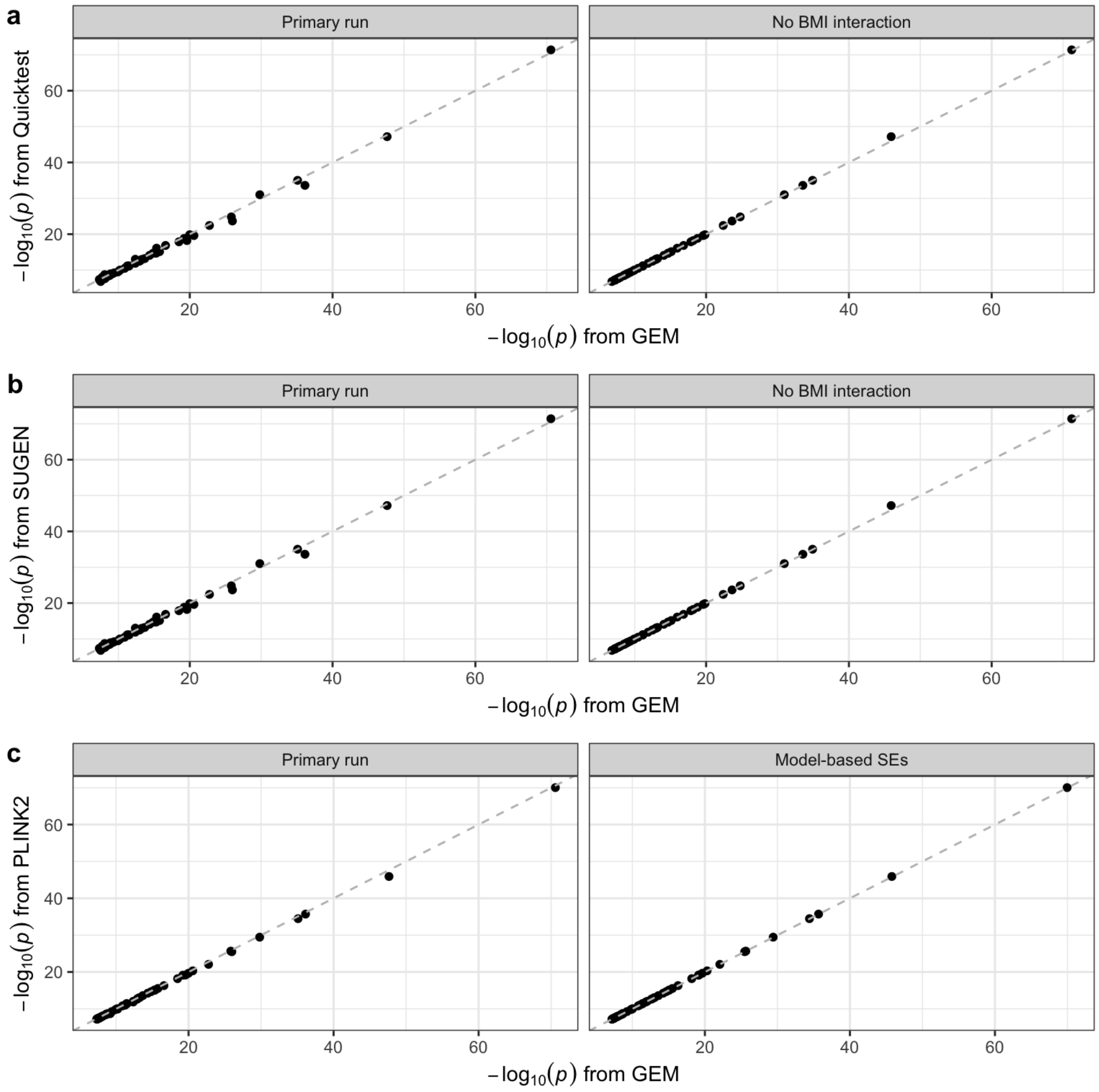

Supplementary Figure S9: Comparison between results from GEM and alternative software programs for top interaction test hits. (a) Negative logarithms of p-values are shown for QUICKTEST (*y*-axis) and GEM (*x*-axis). Because QUICKTEST cannot incorporate interaction covariates, it is compared against GEM p-values from both the primary model (including the BMI interaction covariate; left panel) and a model adjusting for BMI but not including an interaction covariate (equivalent to the QUICKTEST model; right panel). (b) As in (a), but comparing to SUGEN. (c) As in (a), but comparing to PLINK2. As PLINK2 can incorporate interaction covariates but not robust standard errors, the right panel displays results from a GEM run including the BMI interaction covariate and using model-based standard errors.

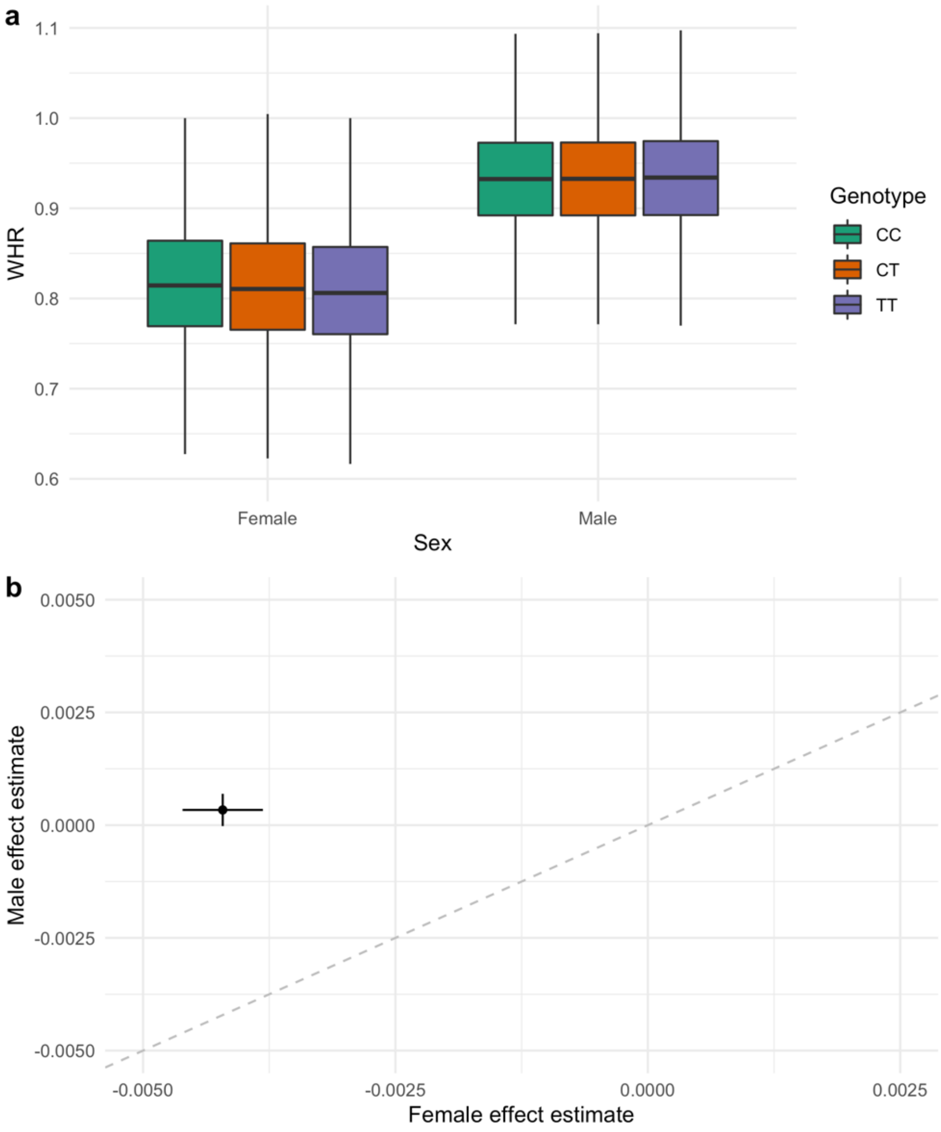

Supplementary Figure S10: Sex dimorphism of the effect of rs13389219 on waist-hip ratio (WHR). (a) Statistical summaries of WHR are shown as a function of sex (primary x-axis) and genotype at rs13389219 (fill colors). Hard-called genotypes were assigned if the corresponding genotype probability was at least 90%, and otherwise the sample was excluded for visualization. Boxplots represent medians (middle bar), first and third quartiles (hinges), and 1.5 interquartile ranges from the hinges (whiskers). Outliers outside of the boxplot whiskers are not shown for ease of visualization. (b) Genetic effect estimates (in raw WHR units) for rs13389219 from sex-stratified analyses (x-axis: females; y-axis: males) are compared. The model was equivalent to that used in the primary analysis other than the removal of interaction terms. Horizontal and vertical bars represent 95% confidence intervals for the female and male effect estimates, respectively, and the dashed line represents $y = x$.

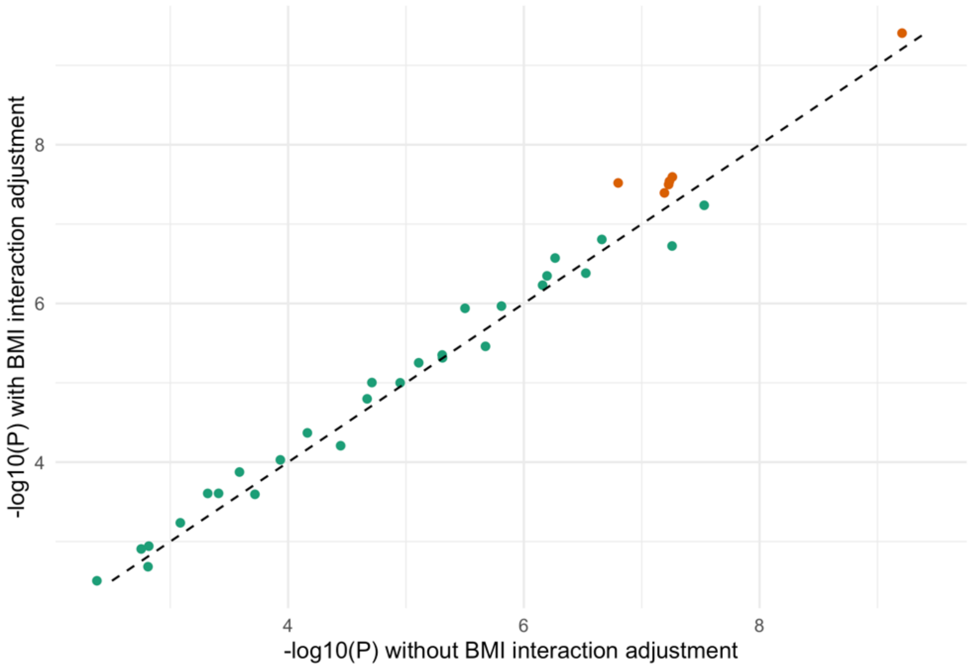

Supplementary Figure S11: Influence of $G \times BMI$ interaction adjustment on $G \times sex$ interaction tests. y- and x-axes display negative logarithms of p-values obtained from the primary model (with a $G \times BMI$ interaction adjustment) and a model without the $G \times BMI$ interaction adjustment, respectively. Variants included in the plot are those found to be sex dimorphic in Pulit et al. (at $p < 5x10^{-8}$) with no genome-wide significant interaction in our analysis (shown in green) or vice versa (shown in orange).

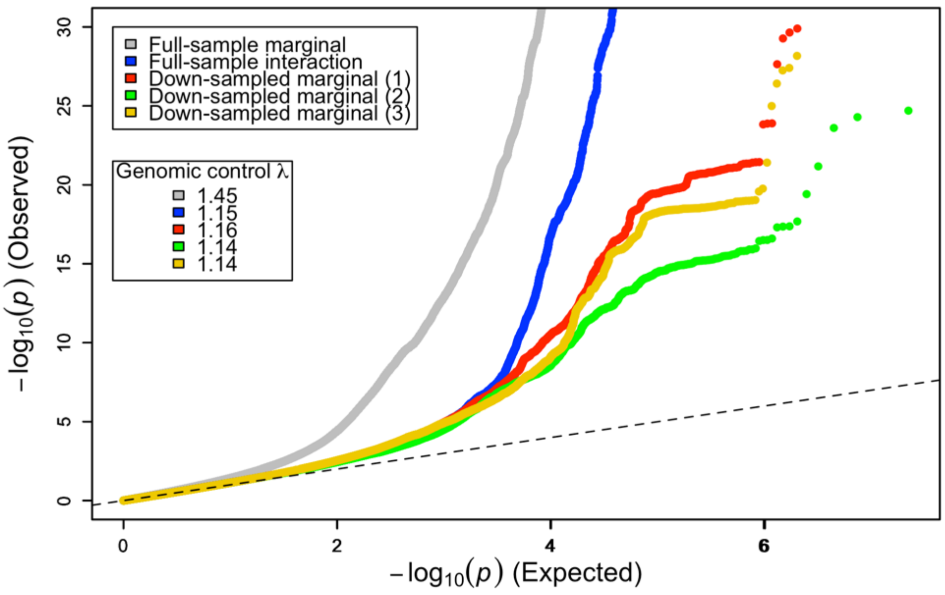

Supplementary Figure S12: Genomic inflation after down-sampling. Genomic inflation lambda values were calculated for the marginal genetic effect terms for each of three random down-samplings of the UK Biobank dataset (see Methods). Quantile-quantile plots show observed versus expected $-\log_{10}(p)$ for either marginal or interaction terms (as labeled). Gray and blue curves correspond to the marginal genetic effect and GEI effect for the full-sample analysis (N=352,768), while red, green, and yellow curves correspond to the marginal genetic effect for the three down-sampled datasets (N=87,695 each). Only p-values > 10^{-30} are shown for the purposes of visualization.

3 Supplementary Tables

Supplementary Table S1: Description of power simulations.

Corresponding panel in Fig. 1	K (active exposures)	q (tested exposures)	G main effect variance explained	Total G×E effect variance explained
A	{1, 2, 5, 10}	{1, 2, 5, 10} (q = K)	0%	Varied*
B	1	{1, 2, 5, 10}	0%	Varied*
C	10	{1, 2, 5, 10}	0%	Varied*
D	1	1	{0%, 0.1%}	Varied*

* For all scenarios, the series of simulated G×E effects was as follows (in terms of percent variance explained): {0.001, 0.0025, 0.005, 0.0075, 0.01, 0.0125, 0.015, 0.0175, 0.02}.

Supplementary Table S2: Genome-wide significant loci from the joint test from the UK Biobank analysis of Waist-Hip Ratio

Chr	Locus	Index SNP	ANNOVAR annotated gene(s)	P	Marginal locus	Pulit et al. main effect locus
6	125915016-128297986	rs577721086	HEY2; NCOA7; HINT3; TRMT11; CENPW; RSPO3; RNF146; ECHDC1; SOGA3; SOGA3; KIAA0408; C6orf58; THEMIS; PTPRK	7.6E-221	Yes	Yes
6	43704572-43829941	rs998584	VEGFA	2E-117	Yes	Yes
2	165205260-165909609	rs13389219	GRB14; COBLL1; SLC38A11	3.2E-102	Yes	Yes
12	122258118-124843104	rs7133378	SETD1B; HPD; PSMD9; RP11-87C12.2; WDR66; BCL7A; MLXIP; LRRC43; IL31; B3GNT4; DIABLO; RP11-512M8.5; VPS33A; CLIP1; ZCCHC8; RSRC2; KNTC1; HCAR1; HCAR2; HCAR3; DENR; CCDC62; HIP1R; VPS37B; ABCB9; OGFOD2; ARL6IP4; PITPNM2; MPHOSPH9; C12orf65; CDK2AP1; SBNO1; SETD8; RILPL2; SNRNP35; RILPL1; TMED2; DDX55; EIF2B1; GTF2H3; TCTN2; ATP6V0A2; DNAH10; CCDC92; DNAH10OS; ZNF664; FAM101A; NCOR2	3.9E-79	Yes	Yes
1	219255260-219931573	rs2605098	LYPLAL1; SLC30A10	9.9E-71	Yes	Yes
3	64641698-65249185	rs4616635	ADAMTS9	2.1E-70	Yes	Yes

6	34013546-35728871	rs201536174	GRM4; HMGA1; C6orf1; NUDT3; RPS10-NUDT3; RPS10; PACSIN1; SPDEF; C6orf106; SNRPC; UHRF1BP1; TAF11; ANKS1A; TCP11; SCUBE3; ZNF76; DEF6; PPARD; FKBP5; ARMC12	2.2E-68	Yes	Yes
12	26252548-26681453	rs718314	BHLHE41; SSPN; ITPR2	6.8E-54	Yes	Yes
1	118849762-119877219	rs6428789	TBX15; WARS2	9E-49	Yes	Yes
1	169768981-170910202	rs3119837	C1orf112; METTL18; SCYL3; METTL11B; GORAB; PRRX1; MROH9	3.2E-48	Yes	Yes
3	128462366-129750377	rs11929498	RAB7A; ACAD9; KIAA1257; RP11-723O4.6; EFCC1; RAB43; ISY1-RAB43; ISY1; CNBP; COPG1; HMCES; H1FX; EFCAB12; MBD4; IFT122; RHO; H1FOO; PLXND1; TMCC1; TRH	4E-44	Yes	Yes
6	6692142-7327846	rs1294415	RREB1; SSR1; CAGE1	6.2E-44	Yes	Yes
4	89457915-90124643	rs2167750	HERC3; NAP1L5; FAM13A; TIGD2	2.7E-43	Yes	Yes
5	55746702-55876283	rs3936510	CTC-236F12.4; AC022431.2	1.3E-41	Yes	Yes
19	33784657-34039207	rs10403360	CEBPA; CEBPG; PEPD	9.9E-39	Yes	Yes
3	156794425-156855232	rs56406311	CCNL1	2.2E-38	Yes	Yes
16	81439073-81550729	rs2925979	CMIP	6.9E-37	Yes	Yes
12	53750337-54685880	rs56154542	CALCOCO1; HOXC13; HOXC12; HOXC10; RP11-834C11.12; HOXC6; HOXC9; HOXC8; HOXC4; HOXC5; HOXC4; SMUG1; HNRNPA1; NFE2; COPZ1	7.1E-36	Yes	Yes
7	25823647-26456537	rs1534696	NFE2L3; HNRNPA2B1; CBX3; SNX10	9E-36	Yes	Yes
20	45454575-45843856	rs2236519	EYA2; ZMYND8	1.3E-35	Yes	Yes
18	2846499-2872586	rs3810068	EMILIN2	1.5E-34	Yes	Yes
17	68204594-68531706	rs2159437		4E-34	Yes	Yes

5	173257052-173593546	rs6897617	CPEB4; C5orf47; NSG2	2.6E-33	Yes	Yes
1	172302544-172463933	rs10752944	DNM3; PIGC; C1orf105	3E-33	Yes	Yes
6	139805476-139844429	rs635769		4.5E-33	Yes	Yes
3	12026479-12859004	rs10602803	TIMP4; PPARG; TSEN2; C3orf83; MKRN2; RAF1; TMEM40; CAND2	4.8E-31	Yes	Yes
19	18179672-18696055	rs7256111	IL12RB1; MAST3; PIK3R2; PIK3R2; IFI30; MPV17L2; RAB3A; PDE4C; KIAA1683; JUND; LSM4; LRRC25; SSBP4; ISYNA1; ELL; FKBP8; UBA52; CRLF1; C19orf60	1.2E-30	Yes	Yes
11	63806993-64185651	rs56271783	MACROD1; FLRT1; STIP1; FERMT3; TRPT1; NUDT22; DNAJC4; VEGFB; FKBP2; PPP1R14B; PLCB3; BAD; GPR137; KCNK4; TEX40; ESRRA; TRMT112; PRDX5; CCDC88B; RPS6KA4	2E-30	Yes	Yes
6	100597579-100629461	rs2073267		1.6E-29	Yes	Yes
13	49540727-51465434	rs1262720	FNDC3A; CDADC1; CAB39L; RCBTB1; ARL11; EBPL; KPNA3; SPRYD7; TRIM13; KCNRG; DLEU1; DLEU7	1.2E-28	Yes	Yes
22	28341062-29454477	rs2294239	TTC28; CHEK2; HSCB; CCDC117; XBP1; ZNRF3; C22orf31	2.1E-25	Yes	Yes
5	176509851-176809618	rs244723	ZNF346; FGFR4; NSD1; RAB24; MXD3; PRELID1; LMAN2; RGS14; SLC34A1	4.1E-25	Yes	Yes
7	130422934-130467575	rs13241165	KLF14	5.4E-25	Yes	Yes
9	107633725-107967832	rs76908150	ABCA1	6.8E-25	Yes	Yes
3	52148623-53556114	rs10933	POC1A; ALAS1; TLR9; TLR9; TWF2; PPM1M; WDR82; GLYCTK; DNAH1; BAP1; PHF7; SEMA3G; TNNC1; NISCH; STAB1; NT5DC2; SMIM4; PBRM1; GNL3; GLT8D1; SPCS1; NEK4; ITIH1; ITIH3; ITIH4; RP5-966M1.6; MUSTN1; TMEM110-MUSTN1; TMEM110; SFMBT1;	8.4E-25	Yes	Yes

			RP11-894J14.5; RFT1; PRKCD; TKT; DCP1A; CACNA1D			
8	72353630-72532296	rs36096231	RP11-1102P16.1	8.2E-24	Yes	Yes
2	110912317-112283722	rs200104212	NPHP1; ACOXL; BCL2L11	1.2E-23	Yes	Yes
3	150018637-150195960	rs62271373	TSC22D2	1.9E-23	Yes	Yes
20	6410854-6635509	rs2145271		4E-22	Yes	Yes
20	33560172-34190870	rs143384	MYH7B; TRPC4AP; EDEM2; PROCR; MMP24; EIF6; FAM83C; UQCC1; GDF5OS; GDF5; CEP250; C20orf173; ERGIC3	8E-22	Yes	Yes
20	5648889-5671460	rs805770		1.3E-21	Yes	Yes
20	51691671-51799439	rs1892203	TSHZ2	1.5E-21	Yes	Yes
11	718436-861814	rs140201358	EPS8L2; TALDO1; PDDC1; AP006621.5; CEND1; SLC25A22; PIDD; RPLP2; PNPLA2; EFCAB4A; CD151; POLR2L; TSPAN4; CHID1	1.3E-20	Yes	Yes
8	25242721-25789968	rs11992444	DOCK5; GNRH1; KCTD9; CDCA2; EBF2	1.6E-20	Yes	Yes
1	154836444-156190363	rs905938	KCNN3; PMVK; PBXIP1; PYGO2; SHC1; CKS1B; FLAD1; LENEP; ZBTB7B; DCST2; DCST1; ADAM15; EFNA4; EFNA3; EFNA3; EFNA1; SLC50A1; DPM3; KRTCAP2; RP11-201K10.3; TRIM46; MUC1; THBS3; MTX1; GBA; FAM189B; SCAMP3; CLK2; HCN3; PKLR; FDPS; RUSC1; ASH1L; MSTO1; YY1AP1; DAP3; GON4L; SYT11; RIT1; KIAA0907; RXFP4; ARHGEF2; SSR2; UBQLN4; LAMTOR2; RAB25; MEX3A; LMNA; SEMA4A; SLC25A44; PMF1-BGLAP; PMF1	3.6E-20	Yes	Yes
2	187718869-188664170	rs13432996	ZSWIM2; CALCRL; TFPI	7.5E-20	Yes	Yes
8	126447308-126616850	8:126511216_GA_G	TRIB1	7.6E-20	Yes	Yes
8	23551520-23617879	rs1561105	NKX2-6	1.1E-19	Yes	Yes

20	38871634-39231118	rs2207132		1.7E-19	Yes	Yes
5	118639270-119062899	rs12234017	TNFAIP8; HSD17B4	3.1E-19	Yes	Yes
11	62048291-62774845	rs7937146	SCGB2A2; SCGB1D4; ASRGL1; SCGB1A1; AHNAK; EEF1G; MIR3654; TUT1; MTA2; EML3; ROM1; B3GAT3; GANAB; INTS5; RP11-831H9.11; C11orf48; METTL12; C11orf83; UBXN1; LRRN4CL; HNRNPUL2-BSCL2; BSCL2; GNG3; HNRNPUL2; TTC9C; STX5; WDR74; SLC22A6; SLC22A8	3.1E-19	Yes	Yes
17	74135619-74293603	rs148951821	FOXJ1; RNF157; UBALD2; QRICH2	4.8E-19	Yes	Yes
7	26928040-27314422	rs17501111	SKAP2; HOXA1; HOXA2; HOXA3; HOXA4; HOXA5; HOXA6; HOXA7; HOXA9; RP1-170O19.20; HOXA10; HOXA11; HOXA13; EVX1	5.6E-19	Yes	Yes
18	60845884-60874413	rs12454712	BCL2	6.5E-19	Yes	Yes
14	91313812-91607703	rs11159989	RPS6KA5; C14orf159	6.8E-19	Yes	Yes
5	141769319-142171097	rs10477191	FGF1; ARHGAP26	2.5E-18	Yes	Yes
11	111318165-111986264	rs138127836	POU2AF1; RP11-794P6.2; BTG4; C11orf88; LAYN; SIK2; PPP2R1B; ALG9; ALG9; FDXACB1; C11orf1; CRYAB; HSPB2; HSPB2-C11orf52; C11orf52; DIXDC1; DLAT; PIH1D2; C11orf57; TIMM8B; SDHD; SDHD	3.7E-18	Yes	Yes
20	62688359-62762014	rs6090040	ZNF512B; SOX18; TCEA2; RGS19; OPRL1; C20orf201; NPBWR2	1.2E-17	Yes	Yes
17	16897328-18267039	rs12449964	MPRIP; PLD6; RP11-45M22.4; FLCN; COPS3; MED9; RASD1; PEMT; RAI1; SREBF1; TOM1L2; LRRC48; ATPAF2; GID4; DRG2; MYO15A; ALKBH5; LLGL1; FLII; MIEF2; TOP3A; SMCR8; SHMT1	1.3E-17	Yes	Yes
11	68817441-69474984	rs11263432	TPCN2; MYEOV; CCND1; ORAOV1	2.1E-17	Yes	Yes
6	160683381-160976620	rs673736	SLC22A2; SLC22A3; LPA	1.2E-16	Yes	Yes

4	4990298-5042928	rs4450871	CYTL1	1.8E-16	Yes	Yes
3	48177829-50829826	rs7429596	CDC25A; CAMP; ZNF589; PLXNB1; CCDC51; TMA7; ATRIP; SHISA5; PFKFB4; UQCRC1; TMEM89; CELSR3; NCKIPSD; IP6K2; PRKAR2A; SLC25A20; ARIH2OS; ARIH2; P4HTM; WDR6; DALRD3; NDUFAF3; IMPDH2; QRICH1; QARS; USP19; LAMB2; CCDC71; KLHDC8B; C3orf84; CCDC36; RP11-3B7.1; C3orf62; USP4; GPX1; RHOA; TCTA; AMT; NICN1; DAG1; BSN; APEH; MST1; RNF123; AMIGO3; GMPPB; IP6K1; CDHR4; FAM212A; UBA7; TRAIP; CAMKV; RBM6; RBM5; SEMA3F; LSMEM2; IFRD2; CACNA2D2; C3orf18; DOCK3	2.7E-16	Yes	Yes
5	54538003-55223929	rs7721054	CCNO; DHX29; SKIV2L2; PPAP2A; SLC38A9; DDX4; IL31RA; AC008914.1; IL6ST	2.9E-16	Yes	Yes
17	43456240-44906949	rs11653367	ARHGAP27; PLEKHM1; CRHR1; SPPL2C; MAPT; STH; KANSL1; ARL17B; LRRC37A; NSF; WNT3; WNT9B	4.9E-16	Yes	Yes
11	65351074-66058612	rs4645917	SSSCA1; FAM89B; EHBP1L1; AP001362.1; KCNK7; MAP3K11; PCNXL3; SIPA1; RELA; KAT5; RNASEH2C; AP5B1; OVOL1; CFL1; SNX32; MUS81; EFEMP2; CTSW; FIBP; SART1; EIF1AD; BANF1; CST6; CATSPER1; PACS1; KLC2; RAB1B; CNIH2; YIF1A; TMEM151A	5.7E-16	Yes	Yes
1	102969517-103757081	rs36000087	COL11A1	7.3E-16	Yes	Yes
1	9320387-9363091	rs72641832	H6PD; SPSB1	9E-16	Yes	Yes
7	71942204-73060006	rs55747707	POM121; TRIM74; TRIM50; FKBP6; FZD9; BAZ1B; BCL7B; TBL2; MLXIPL	1.2E-15	Yes	Yes
12	66426835-66452879	rs11176019		1.5E-15	Yes	Yes
10	122807950-122917290	rs2254069		2.5E-15	Yes	Yes

4	26005926-26491311	rs7695004	RBPJ; CCKAR	2.7E-15	Yes	Yes
6	25413403-29612325	rs9257133	LRRC16A; SCGN; HIST1H2AA; HIST1H2BA; SLC17A4; SLC17A1; SLC17A3; SLC17A2; TRIM38; HIST1H1A; HIST1H3A; HIST1H4A; HIST1H4B; HIST1H3B; HIST1H2AB; HIST1H2BB; HIST1H3C; HIST1H1C; HFE; HIST1H4C; HIST1H1T; HIST1H2BC; HIST1H2AC; HIST1H1E; HIST1H2BD; HIST1H2BE; HIST1H4D; HIST1H3D; HIST1H2AD; HIST1H2BF; HIST1H4E; HIST1H3E; HIST1H1D; HIST1H3G; HIST1H2BI; BTN3A2; BTN2A2; BTN3A1; BTN3A3; BTN2A1; BTN1A1; HMGN4; ABT1; ZNF322; HIST1H2BJ; HIST1H2AG; HIST1H2BK; HIST1H4I; HIST1H2AH; PRSS16; POM121L2; ZNF391; ZNF184; HIST1H2BL; HIST1H2AI; HIST1H3H; HIST1H2AJ; HIST1H2BM; HIST1H4J; HIST1H4K; HIST1H2AK; HIST1H2BN; HIST1H2AL; HIST1H1B; HIST1H3I; HIST1H4L; HIST1H3J; HIST1H2AM; HIST1H2BO; OR2B2; OR2B6; ZNF165; ZSCAN16; ZKSCAN8; ZSCAN9; ZKSCAN4; NKAPL; PGBD1; ZSCAN31; ZKSCAN3; ZSCAN12; ZSCAN23; GPX6; GPX5; SCAND3; TRIM27; C6orf100; ZNF311; OR2W1; OR2B3; OR2J1; OR2J3; OR2J2; OR14J1; OR5V1; OR12D3; OR12D2; OR11A1; OR10C1; OR2H1; MAS1L; UBD; GABBR1; OR2H2	3E-15	Yes	No
6	80693816-81922230	rs1902066	TTK; BCKDHB	3.4E-15	Yes	Yes
17	40228564-41455740	rs72823057	CTD-2132N18.3; RAB5C; KCNH4; STAT5B; STAT5A; STAT3; PTRF; ATP6V0A1; NAGLU; HSD17B1; COASY; MLX; PSMC3IP; FAM134C; TUBG1; TUBG2; PLEKHH3; CCR10; CNTNAP1; EZH1; RAMP2; VPS25; WNK4; COA3; CNTD1; BECN1; PSME3;	3.4E-15	Yes	Yes

			AOC2; G6PC; RPL27; IFI35; VAT1; NBR1			
4	56148989-56534563	rs10462028	SRD5A3; TMEM165; CLOCK; PDCL2; NMU	4.2E-15	Yes	Yes
5	132146848-132466540	rs55747751	SEPT8; SOWAHA; SHROOM1; ZCCHC10; HSPA4	4.4E-15	Yes	Yes
7	77112367-77606013	rs558036185	PTPN12; RSBN1L; TMEM60; PHTF2	6.4E-15	Yes	Yes
8	128273489-128401772	rs378854		7.5E-15	Yes	Yes
2	66129494-66326994	rs6719428		8.7E-15	Yes	Yes
4	145227600-146174823	rs200457388	HHIP; ANAPC10; ABCE1; OTUD4	1.2E-14	Yes	Yes
16	4277445-4596447	rs7200336	SRL; TFAP4; GLIS2; PAM16; CORO7-PAM16; CORO7; VASN; DNAJA3; NMRAL1; HMOX2; CDIP1	2.1E-14	Yes	Yes
7	28138193-28256240	rs849134	JAZF1	2.2E-14	Yes	Yes
10	4829609-5061978	rs66923674	AKR1E2; AKR1C1; AKR1C2	2.3E-14	Yes	Yes
3	137906532-138165540	rs9872754	ARMC8; NME9; MRAS; ESYT3	2.7E-14	Yes	Yes
10	114729482-114823426	rs4073980	TCF7L2	2.8E-14	Yes	Yes
7	80549535-80609231	rs917191	SEMA3C	3.5E-14	Yes	Yes
2	13071963-13111388	rs779390		3.6E-14	Yes	Yes
5	3944907-4101569	rs11134029		3.6E-14	Yes	Yes
17	59471082-59546366	rs757608	BCAS3; TBX2; C17orf82; TBX4	4.5E-14	Yes	Yes
8	8088933-11895484	rs2980755	SGK223; CLDN23; MFHAS1; ERI1; PPP1R3B; RP11-10A14.4; TNKS; MSRA; PRSS55; RP1L1; C8orf74; SOX7; SOX7; PINX1; XKR6; AF131215.5; MTMR9; SLC35G5; C8orf12; FAM167A; BLK; GATA4; C8orf49; NEIL2; FDFT1; RP11-297N6.4; CTSB; DEFB136; DEFB135; DEFB134; RP11-481A20.11	7E-14	Yes	Yes

1	77911973-78870285	rs140681455	AK5; ZZZ3; USP33; FAM73A; NEXN; FUBP1; DNAJB4; GIPC2; PTGFR	1E-13	No	No
19	45386467-45427125	rs429358	PVRL2; TOMM40; APOE; APOC1	1.5E-13	Yes	Yes
12	9044440-9268585	rs1805741	A2ML1; PHC1; M6PR; KLRG1; A2M	2.1E-13	Yes	Yes
12	47842589-48202696	rs145878042	RAPGEF3; SLC48A1; HDAC7	2.3E-13	Yes	Yes
4	699217-1013634	rs13101828	PCGF3; CPLX1; GAK; TMEM175; DGKQ; SLC26A1; IDUA; FGFRL1	2.5E-13	Yes	Yes
9	94932390-95641226	rs754600	IARS; NOL8; CENPP; OGN; OMD; ASPN; ECM2; IPPK; BICD2; ZNF484	2.8E-13	Yes	Yes
14	58643368-59071574	rs111735080	C14orf37; ACTR10; PSMA3; AL132989.1; ARID4A; TOMM20L; TIMM9; KIAA0586	2.9E-13	Yes	Yes
7	107607970-107643977	rs77775720	LAMB1	3.7E-13	Yes	Yes
2	67697936-67869772	rs6721459		4.2E-13	Yes	Yes
10	95309022-95359494	rs12241416	FFAR4; RBP4	5.6E-13	Yes	Yes
17	7321858-7559677	rs858519	NLGN2; SPEM1; C17orf74; TMEM102; FGF11; CHRNB1; ZBTB4; SLC35G6; POLR2A; TNFSF12; TNFSF12-TNFSF13; TNFSF13; SENP3; EIF4A1; CD68; MPDU1; SOX15; FXR2; AC007421.1; SHBG; SAT2; ATP1B2; TP53	5.6E-13	Yes	Yes
2	158282978-158762831	rs55920843	CYTIP; ACVR1C; ACVR1; UPP2	6.1E-13	Yes	Yes
6	43084747-43414234	rs35121648	PTK7; CUL9; DNPH1; TTBK1; SLC22A7; CRIP3; ZNF318; ABCC10; DLK2	1.3E-12	Yes	No
10	80907147-81018948	rs779933	ZMIZ1	1.6E-12	Yes	Yes
21	46764460-46807499	rs759304654		1.7E-12	Yes	Yes
15	56350033-56785485	rs140739203	RFX7; TEX9; MNS1	2.6E-12	Yes	Yes
19	7076791-7231991	rs1799815	ZNF557; INSR	2.7E-12	Yes	Yes

17	27653448-27912109	rs62070804	TAOK1; ABHD15; TP53I13; GIT1; ANKRD13B	3.7E-12	Yes	Yes
17	73075643-73538318	rs2385263	SLC16A5; NUP85; GGA3; MRPS7; MIF4GD; SLC25A19; GRB2; KIAA0195; CASKIN2; TSEN54; LLGL2	4.1E-12	Yes	Yes
9	113831319-114044933	rs10980797		4.7E-12	Yes	Yes
4	102702364-103387160	rs13107325	BANK1; SLC39A8	5.8E-12	Yes	Yes
10	63699921-64044448	rs9415646	ARID5B; RTKN2	7.3E-12	Yes	Yes
16	49847939-49881726	rs34050011	ZNF423	7.6E-12	Yes	Yes
15	31631269-31730442	rs8027155	KLF13	7.9E-12	Yes	Yes
15	41801512-42212374	rs4923914	ITPKA; LTK; RPAP1; TYRO3; MGA; MAPKBP1; AC073657.1; PLA2G4B; JMJD7; JMJD7-PLA2G4B; SPTBN5; EHD4	8.5E-12	Yes	Yes
2	216235538-216316487	rs3910516	FN1	8.6E-12	Yes	Yes
11	9827871-10426129	rs7104821	SBF2; ADM; AMPD3	9.6E-12	Yes	Yes
12	45968598-45995155	rs73108788		1.2E-11	Yes	Yes
1	220967854-221428183	rs1935157	MARC2; MARC1; HLX	1.2E-11	Yes	Yes
1	2898494-3002692	rs2993481	ACTRT2; PRDM16	1.2E-11	Yes	Yes
3	46925539-47583156	rs4682844	MYL3; PTH1R; AC109583.1; CCDC12; NBEAL2; SETD2; KIF9; KLHL18; PTPN23; SCAP; ELP6	1.5E-11	Yes	No
2	172252999-172479885	2:172408827_CA_C	METTL8; DCAF17; CYBRD1	1.9E-11	Yes	Yes
8	135598132-135790049	rs7834111	ZFAT	2.6E-11	Yes	Yes
19	55985786-56009191	rs8103017	ZNF628; NAT14; SSC5D	2.8E-11	Yes	Yes
3	185459675-185548683	rs558655224	IGF2BP2	2.8E-11	Yes	Yes
8	89191810-89761163	rs7014590	MMP16	3E-11	Yes	Yes

3	11227421-11688393	rs201740704	HRH1; ATG7; VGLL4	3E-11	Yes	Yes
4	123055360-124312571	rs303084	KIAA1109; ADAD1; BBS12; FGF2; NUDT6; SPATA5; SPRY1	5E-11	Yes	Yes
2	218329039-218393389	rs7579468	DIRC3	5.1E-11	Yes	Yes
2	25162667-25729690	rs13032289	DNAJC27; EFR3B; POMC; DNMT3A; DTNB	5.3E-11	Yes	Yes
21	39391088-39723775	21:39509490_CAA_C	DSCR4; DSCR8; KCNJ15	5.9E-11	Yes	Yes
20	569164-645733	rs144033177	SRXN1; RP5-850E9.3; SCRT2	6E-11	Yes	Yes
3	171759410-171829456	rs4894803	FNDC3B	8.4E-11	Yes	Yes
19	17152232-17230199	rs1077795	HAUS8; MYO9B	8.6E-11	Yes	Yes
6	133447707-133663743	rs149692566	EYA4	1.1E-10	Yes	Yes
11	47229316-48335505	11:47428209_TA_T	DDB2; ACP2; NR1H3; MADD; MYBPC3; SPI1; SLC39A13; PSMC3; RAPSN; CELF1; NDUFS3; PTPMT1; KBTBD4; KBTBD4; FAM180B; C1QTNF4; MTCH2; AGBL2; FNBP4; NUP160; PTPRJ; OR4S1	1.2E-10	Yes	Yes
16	66776186-68407575	rs8052655	DYNC1LI2; CCDC79; NAE1; CDH16; RRAD; FAM96B; CES2; CES3; CES4A; CBFB; C16orf70; B3GNT9; TRADD; FBXL8; HSF4; RP11-5A19.5; KIAA0895L; EXOC3L1; E2F4; ELMO3; LRRC29; AC040160.1; TMEM208; FHOD1; SLC9A5; PLEKHG4; KCTD19; LRRC36; TPPP3; ZDHHC1; HSD11B2; ATP6V0D1; AGRP; FAM65A; CTCF; RLTPR; ACD; PARD6A; ENKD1; C16orf86; GFOD2; RANBP10; TSNAXIP1; CENPT; THAP11; NUTF2; EDC4; NRN1L; PSKH1; CTRL; CTC-479C5.12; PSMB10; LCAT; SLC12A4; DPEP3; DPEP2; DUS2; DDX28; NFATC3; ESRP2; PLA2G15; SLC7A6; SLC7A6OS; PRMT7; SMPD3	1.3E-10	Yes	Yes
2	171342637-171421606	rs781693294	MYO3B	1.4E-10	Yes	Yes

2	119307659-119572451	rs332105		1.5E-10	Yes	Yes
6	130321899-130459410	rs13211683	L3MBTL3; SAMD3	1.5E-10	Yes	Yes
11	36285995-36391882	rs112013938	COMMD9; PRR5L	1.8E-10	Yes	Yes
7	116879224-117382261	rs2188555	ST7; WNT2; ASZ1; CFTR; CTTNBP2	2E-10	Yes	Yes
12	133682655-133810286	rs11147235	ZNF140; ZNF891; ZNF10; ZNF268; CTD-2140B24.4; AC226150.4; ANHX	2.1E-10	Yes	Yes
1	22602254-22633113	rs140800754		2.1E-10	Yes	Yes
10	21706004-22298641	rs7084454	CASC10; SKIDA1; MLLT10; DNAJC1	2.8E-10	No	No
17	79897449-80023991	rs35344256	PYCR1; MYADML2; NOTUM; ASPSCR1; STRA13; LRRC45; RAC3; DCXR; RFNG; GPS1; DUS1L	2.8E-10	Yes	Yes
2	114121700-114804521	rs757793886	CBWD2; AC016745.1; FOXD4L1; RABL2A; SLC35F5; ACTR3	3E-10	Yes	Yes
20	50817889-51264331	rs36119055	ZFP64	3E-10	Yes	Yes
14	52149643-52695513	rs140664623	FRMD6; GNG2; AL358333.1; C14orf166; NID2	3.2E-10	Yes	Yes
10	34160764-34197590	rs10763957		3.2E-10	Yes	Yes
6	14515031-14617591	rs6932767		3.3E-10	Yes	Yes
7	104579775-105088693	7:104885481_AACACAC_A	KMT2E; SRPK2; PUS7	3.6E-10	Yes	Yes
5	180644299-180714439	rs11746028	TRIM41; GNB2L1; TRIM52; AC008443.1	4E-10	Yes	Yes
15	40737490-41126473	rs8036817	IVD; BAHD1; CHST14; C15orf57; RPUSD2; CASC5; RAD51; RMDN3; GCHFR; DNAJC17; C15orf62; ZFYVE19; PPP1R14D; SPINT1	4.3E-10	Yes	No
11	14138211-14936943	rs76613195	RRAS2; COPB1; PSMA1; PSMA1; PDE3B; CYP2R1; CALCB	4.4E-10	Yes	Yes
18	20680619-20834307	18:20737641_TTAA_T	CABLES1; TMEM241	4.9E-10	Yes	Yes
12	106960182-107375551	rs1922432	RP11-144F15.1; RFX4; RIC8B; C12orf23; MTERFD3; CRY1	5E-10	Yes	Yes

2	206826211-207208563	rs11893623	INO80D; NDUFS1; EEF1B2; GPR1; ZDBF2	5E-10	Yes	Yes
5	33991129-34049157	rs299615	SLC45A2; AMACR; RP11-1084J3.4; C1QTNF3	5.3E-10	Yes	Yes
18	46553383-47097372	rs112081515	DYM; C18orf32; RPL17-C18orf32; RPL17; LIPG	6.3E-10	Yes	Yes
2	66726568-66806603	rs13432332	MEIS1	6.6E-10	Yes	Yes
11	13257150-13356159	11:13356159_TAGA_T	ARNTL	7.5E-10	Yes	No
22	30236261-30997745	rs13056562	ASCC2; HORMAD2; LIF; OSM; GATSL3; RP1-130H16.18; TBC1D10A; SF3A1; CCDC157; RNF215; SEC14L2; RP4-539M6.19; KIAA1658; MTFP1; SEC14L3; SEC14L4; SEC14L6; GAL3ST1; PES1; TCN2	7.6E-10	Yes	No
3	78672360-79013878	rs4681011	ROBO1	7.9E-10	Yes	Yes
5	171779917-171876084	rs6893600	SH3PXD2B	8.3E-10	Yes	Yes
20	39547593-40243126	rs4812492	TOP1; PLCG1; ZHX3; LPIN3; EMILIN3; CHD6	8.4E-10	Yes	Yes
7	101633768-101892833	rs421168	CUX1	9.6E-10	Yes	Yes
10	119245385-119348243	rs4752082	EMX2	1.2E-09	Yes	Yes
10	27674855-27967634	rs1494204	PTCHD3; RAB18; MKX	1.2E-09	Yes	Yes
17	70247400-70316434	rs7211132		1.3E-09	Yes	Yes
1	65506317-65593558	rs6588110		1.4E-09	Yes	No
6	41666065-41848061	rs35629562	TFEB; PGC; FRS3; PRICKLE4; TOMM6; USP49	1.4E-09	Yes	Yes
12	33510115-34730073	rs10844642	SYT10; ALG10	1.5E-09	Yes	Yes
11	32294884-32551861	rs7105538	WT1	1.5E-09	Yes	No
4	106045245-106448002	rs78632895	TET2; PPA2	1.5E-09	Yes	Yes
17	53601168-53738843	rs919134		1.6E-09	Yes	Yes

3	35433169-35906275	rs9818103	ARPP21	1.7E-09	Yes	Yes
1	204979752-205367233	rs6593925	NFASC; CNTN2; TMEM81; RBBP5; DSTYK; TMCC2; NUAK2; KLHDC8A; LEMD1	1.8E-09	Yes	Yes
3	168909613-168974215	rs998749	MECOM	1.9E-09	Yes	Yes
8	69514642-69657438	rs12543555	C8orf34	2E-09	Yes	Yes
5	101553994-102834360	rs755015434	SLCO4C1; AC008948.1; SLCO6A1; PAM; GIN1; PPIP5K2; C5orf30	2.1E-09	Yes	Yes
15	51061883-51643333	rs727479	SPPL2A; AP4E1; TNFAIP8L3; CYP19A1; GLDN	2.2E-09	Yes	Yes
19	2129473-2226676	rs4806832	AP3D1; DOT1L; PLEKHJ1; SF3A2	2.3E-09	Yes	Yes
10	32516911-32816350	rs3740237	EPC1; CCDC7	2.3E-09	Yes	Yes
4	119989522-120700284	rs10025536	SYNPO2; MYOZ2; RP11-455G16.1; USP53; C4orf3; FABP2; PDE5A	2.4E-09	No	Yes
5	66278913-66505010	rs4016246	MAST4; CD180	2.4E-09	Yes	Yes
13	110906034-111080609	rs1999013	COL4A1; COL4A2	2.8E-09	Yes	Yes
2	100205498-100543169	rs12620982	AFF3	3E-09	Yes	Yes
16	11618937-11645198	rs57792815	CTD-3088G3.8; LITAF	3.5E-09	No	Yes
8	71464015-72012957	rs17697852	TRAM1; AC120194.1; RP11-382J12.1; LACTB2; XKR9	3.6E-09	Yes	Yes
6	85038095-85574238	rs36194565	TBX18	3.9E-09	Yes	Yes
7	42535610-42762246	rs13230133		3.9E-09	Yes	Yes
22	38510258-38649783	rs2277844	BAIAP2L2; PLA2G6; MAFF; TMEM184B	4E-09	No	No
1	203504544-203538486	rs13303359		4.4E-09	No	Yes
12	102394872-102946220	rs11111146	DRAM1; CCDC53; NUP37; PARPBP; PMCH; IGF1	4.5E-09	No	Yes
16	53395441-53567931	rs11540358	RBL2; AKTIP	4.5E-09	Yes	Yes

15	67468525-68202469	rs778984966	SMAD3; AAGAB; IQCH; C15orf61; MAP2K5; SKOR1	4.9E-09	No	Yes
6	153303253-153529146	6:153429671_AT_A	FBXO5; MTRF1L; RGS17	5.3E-09	Yes	No
17	54741088-54795135	rs227732		5.5E-09	Yes	Yes
11	26046964-26363333	rs11605956	ANO3	5.9E-09	Yes	Yes
9	112528882-112595175	rs2209815	PALM2; PALM2-AKAP2; AKAP2	6.9E-09	Yes	Yes
12	113479223-113522774	rs10850127	DTX1	7E-09	Yes	No
5	134398345-134412802	rs140814212	C5orf66	7.3E-09	No	No
2	48956431-49001083	rs17326656	STON1-GTF2A1L; GTF2A1L; LHCGR	8.3E-09	No	Yes
21	35566316-35682958	rs28451064		8.4E-09	No	Yes
1	212354412-212636985	rs381204	PPP2R5A; TMEM206; NENF	1E-08	Yes	Yes
17	76365791-76442152	rs691094	SOCS3; PGS1; DNAH17; AC061992.1	1E-08	Yes	Yes
18	42407476-42525509	rs635469	SETBP1	1E-08	Yes	Yes
15	100080644-100275110	rs11634364	MEF2A; LYSMD4; DKFZP779J2370	1.1E-08	Yes	Yes
5	75867877-76117836	rs76525389	IQGAP2; F2RL2; F2R; F2RL1	1.3E-08	Yes	No
5	157745474-158048398	5:158008474_TA_T		1.3E-08	No	Yes
3	61487198-61559771	rs7621604	PTPRG	1.3E-08	Yes	Yes
7	30753307-30936024	rs12112380	INMT; INMT-FAM188B; FAM188B; AQP1; AQP1	1.4E-08	No	No
12	37856814-39440577	rs7314177	ALG10B; CPNE8	1.4E-08	Yes	Yes
2	160408763-160894930	2:160731357_TAA_T	BAZ2B; MARCH7; CD302; LY75; LY75-CD302; PLA2R1	1.5E-08	No	No
17	61590481-62020866	rs2854152	ACE; ACE; KCNH6; DCAF7; MAP3K3; LIMD2; STRADA; RP11-51F16.8; CCDC47; DDX42; FTSJ3; PSMC5; SMARCD2; CSH2; GH2; CSH1; CSHL1; GH1; CD79B; SCN4A	1.5E-08	Yes	Yes

3	99310218-100193269	rs147852464	COL8A1; CMSS1; FILIP1L; TMEM30C	1.5E-08	Yes	Yes
2	239640387-239753610	rs11676305	TWIST2	1.6E-08	Yes	Yes
9	93894823-94159364	rs73650963	AUH	1.6E-08	Yes	Yes
12	94077280-94201279	rs10745659	CRADD	1.6E-08	No	Yes
21	36612241-36835576	rs73197345	RUNX1	1.6E-08	Yes	Yes
14	98342603-98486406	rs10130842	C14orf64	1.7E-08	No	Yes
9	101734465-101787017	rs10988442	COL15A1	1.7E-08	Yes	Yes
17	1612199-1657899	rs148325412	TLCD2; WDR81; SERPINF2; SERPINF1	1.8E-08	Yes	Yes
10	104220301-104543883	rs28408682	C10orf95; TMEM180; ACTR1A; SUFU; TRIM8; ARL3; SFXN2; WBP1L	1.9E-08	No	Yes
7	120719961-121093671	rs1534520	CPED1; WNT16	1.9E-08	Yes	Yes
1	11826085-11936514	rs56153133	C1orf167; MTHFR; CLCN6; NPPA; NPPB	2.1E-08	No	Yes
7	93082708-93323303	rs2283006	CALCR; GNGT1	2.1E-08	Yes	Yes
2	57941092-58756729	rs113257513	VRK2; FANCL	2.1E-08	No	Yes
5	59823118-60850522	rs12523278	PDE4D; DEPDC1B; ELOVL7; ERCC8; NDUFAF2; AC008498.1; SMIM15; ZSWIM6	2.2E-08	Yes	No
5	3544272-3601924	rs2166365	IRX1	2.2E-08	Yes	Yes
15	74245007-74333413	rs11072467	LOXL1; STOML1; PML	2.3E-08	Yes	Yes
7	20371758-20480171	rs766298290	ITGB8	2.4E-08	Yes	Yes
12	108514301-108631047	rs3764002	WSCD2	2.7E-08	Yes	Yes
11	10884431-10947836	rs7932891	ZBED5	2.7E-08	Yes	Yes
19	41148101-41323482	rs2042935	NUMBL; ADCK4; ITPKC; C19orf54; SNRPA; MIA-RAB4B; MIA; RAB4B; RAB4B-EGLN2; EGLN2; CTC-490E21.12	2.8E-08	No	No

12	27995650-28730688	rs180958337	CCDC91	2.8E-08	Yes	Yes
5	172130320-172169257	rs322396		2.9E-08	Yes	Yes
14	60786981-61406339	rs1254319	C14orf39; SIX6; SIX1; SIX4; MNAT1	3E-08	Yes	No
1	86251836-86452815	rs313732	COL24A1	3E-08	Yes	Yes
1	200014790-200061056	rs12131072	NR5A2	3E-08	Yes	Yes
3	100910127-101550022	rs62280667	IMPG2; SENP7; TRMT10C; PCNP; CEP97; NXPE3; NFKBIZ	3E-08	No	Yes
12	98976915-99129497	rs9668110	SLC25A3; IKBIP; APAF1; ANKS1B	3.1E-08	No	No
11	116528202-116951533	rs180378	BUD13; ZNF259; APOA5; APOA4; APOC3; APOA1; SIK3	3.4E-08	Yes	No
5	172730637-172748501	rs3836828	STC2	4.1E-08	No	No
16	10924773-10992715	rs12931265	CIITA	4.2E-08	Yes	Yes
1	150521099-150973407	rs200492635	ADAMTSL4; AL356356.1; ADAMTSL4-AS1; MCL1; ENSA; GOLPH3L; HORMAD1; CTSS; CTSK; ARNT; SETDB1; CERS2; ANXA9; FAM63A; PRUNE	4.2E-08	No	Yes
2	226813902-227046342	rs11895712		4.3E-08	No	Yes
17	65331851-65422840	rs8866	PSMD12; PITPNC1	4.3E-08	Yes	Yes
9	96701663-96894266	rs5014099	BARX1; PTPDC1	4.3E-08	Yes	Yes
10	33464462-33509513	rs734187	NRP1	4.3E-08	Yes	Yes
7	135050259-135363998	rs4728358	CNOT4; NUP205; C7orf73; SLC13A4	4.5E-08	No	No
10	95896807-95949818	rs2797985	PLCE1	4.5E-08	No	Yes
9	111647764-111978049	rs12684047	IKBKAP; FAM206A; CTNNAL1; TMEM245; FRRS1L; EPB41L4B	4.7E-08	No	Yes
6	167346852-167541258	rs9459839	RP11-514O12.4; RNASET2; FGFR1OP; CCR6	4.8E-08	No	Yes
5	148549111-148646866	rs35624365	ABLIM3; AFAP1L1	4.9E-08	Yes	Yes

Supplementary Table S3: Genome-wide significant loci from the interaction test from the UK Biobank analysis of Waist-Hip Ratio

Chr	Locus	Index SNP	ANNOVAR annotated gene(s)	P	Marginal locus	Pulit et al. interaction locus
2	164738239-166074183	rs13389219	GRB14; COBLL1; SLC38A11; SCN3A	2.6E-71	Yes	Yes
6	126187585-127762588	rs72959041	NCOA7; RSPO3; RNF146; SOGA3; SOGA3; KIAA0408	2.3E-48	Yes	Yes
1	218684226-219798632	rs11118310	C1orf143; LYPLAL1	7.4E-37	Yes	Yes
6	43756863-43804571	rs4711750	VEGFA	8E-36	Yes	Yes
12	122227152-124812706	rs7978610	TMEM120B; RHOF; SETD1B; HPD; PSMD9; RP11-87C12.2; WDR66; BCL7A; MLXIP; LRRC43; IL31; B3GNT4; DIABLO; RP11-512M8.5; VPS33A; CLIP1; ZCCHC8; RSRC2; KNTC1; HCAR1; HCAR2; HCAR3; DENR; CCDC62; HIP1R; VPS37B; ABCB9; OGFOD2; ARL6IP4; PITPNM2; MPHOSPH9; C12orf65; CDK2AP1; SBNO1; SETD8; RILPL2; RILPL1; TMED2; DDX55; EIF2B1; GTF2H3; TCTN2; ATP6V0A2; DNAH10; CCDC92; DNAH10OS; ZNF664; FAM101A; NCOR2	1.6E-30	Yes	Yes
12	26106056-26612678	rs718314	RASSF8; BHLHE41; SSPN; ITPR2	1.1E-26	Yes	Yes
3	64674272-64861470	rs11130982	ADAMTS9	1.5E-26	Yes	Yes
5	55794632-55877238	rs3936510	AC022431.2	1.7E-23	Yes	Yes
16	81486176-81551482	rs2925979	CMIP	2.6E-21	Yes	Yes
7	130422934-130467575	rs13241165	KLF14	1E-20	Yes	Yes
4	89457915-90049495	rs34154818	HERC3; NAP1L5; FAM13A; TIGD2	2.5E-20	Yes	Yes
3	128755208-129706148	rs9866653	EFCC1; GP9; RAB43; ISY1-RAB43; ISY1; CNBP; COPG1;	6.1E-20	Yes	Yes

			EFCAB12; MBD4; IFT122; RHO; H1FOO; PLXND1; TMCC1; TRH			
17	68257258-68613765	rs9890689		3.3E-19	Yes	Yes
6	139805476-139870189	rs632057		2.5E-17	Yes	Yes
3	12026207-13067845	rs10602803	TIMP4; PPARG; TSEN2; C3orf83; MKRN2; RAF1; TMEM40; IQSEC1	1.7E-16	Yes	Yes
6	34067732-35173158	rs115177000	GRM4; HMGA1; C6orf1; NUDT3; RPS10-NUDT3; RPS10; PACSIN1; SPDEF; C6orf106; SNRPC; UHRF1BP1; TAF11; ANKS1A; SCUBE3	4.5E-16	Yes	Yes
7	25724429-26440503	rs1534696	NFE2L3; SNX10	5.1E-16	Yes	Yes
18	2846499-2873060	rs3810068	EMILIN2	1.4E-15	Yes	Yes
5	118405453-119062899	rs12234017	DMXL1; TNFAIP8; HSD17B4; FAM170A	4.3E-15	Yes	Yes
1	77557339-79055729	rs140681455	PIGK; AK5; ZZZ3; USP33; FAM73A; NEXN; FUBP1; DNAJB4; GIPC2; PTGFR	2E-14	No	Yes
8	126447308-126616850	8:126480512_TTTTTGAGATGCACCCATGTTTTTGTGTATACTAGCATC_T	TRIB1	5.5E-14	Yes	Yes
1	170179930-170791715	rs2104912	GORAB; PRRX1	9.5E-14	Yes	Yes
6	100597579-100629461	rs2073267		1.3E-13	Yes	Yes
3	150018637-150350593	rs62271373	TSC22D2; SERP1; EIF2A; SELT	4.2E-13	Yes	Yes
1	172302544-172454826	rs1052256	DNM3; PIGC; C1orf105	4.3E-13	Yes	Yes
18	60845884-60878883	rs12454712	BCL2	3.2E-12	Yes	Yes
8	23551328-23617879	rs11986562	NKX2-6	3.6E-12	Yes	Yes
9	107676758-107782100	rs10991417	ABCA1	5.6E-12	Yes	Yes

2	111808175-112024305	rs374722324	ACOXL; BCL2L11	1.3E-11	Yes	Yes
11	63509742-64469428	rs71468663	RTN3; C11orf84; MARK2; MACROD1; FLRT1; STIP1; FERMT3; TRPT1; NUDT22; DNAJC4; VEGFB; FKBP2; PPP1R14B; PLCB3; BAD; GPR137; KCNK4; TEX40; ESRRA; TRMT112; PRDX5; CCDC88B; RPS6KA4; SLC22A11; NRXN2	5.9E-11	Yes	Yes
7	80549535-80610777	rs2015768	SEMA3C	6.9E-11	Yes	Yes
4	4990298-5010193	rs4450871	CYTL1	1.1E-10	Yes	Yes
6	6724416-6783937	rs4960246		3.9E-10	Yes	No
20	39142516-39267671	rs117113213		6.3E-10	Yes	Yes
10	95277745-95359494	rs11187535	CEP55; FFAR4; RBP4	1.3E-09	Yes	Yes
20	62688359-62775183	rs6090040	ZNF512B; SOX18; TCEA2; RGS19; OPRL1; C20orf201; NPBWR2; MYT1	1.6E-09	Yes	Yes
22	38432277-38655906	rs2277844	POLR2F; PICK1; SLC16A8; BAIAP2L2; PLA2G6; MAFF; TMEM184B	2.2E-09	No	Yes
8	128273489-128396316	rs424281		2.5E-09	Yes	Yes
10	63795446-63960611	rs4948502	ARID5B; RTKN2	2.6E-09	Yes	Yes
20	51691671-51740178	rs1892204	TSHZ2	4.9E-09	Yes	Yes
6	167345772-167541258	rs9459839	RP11-514O12.4; RNASET2; FGFR1OP; CCR6	8.3E-09	No	Yes
21	46763618-46818782	rs759304654	COL18A1	8.3E-09	Yes	Yes
5	54347571-55313388	rs7735689	GZMA; CDC20B; MCIDAS; CCNO; DHX29; SKIV2L2; PPAP2A; SLC38A9; DDX4; IL31RA; AC008914.1; IL6ST	8.4E-09	Yes	Yes
1	203504544-203568813	rs13303359		1.2E-08	No	Yes
17	27404749-28523314	rs62070804	MYO18A; TIAF1; CRYBA1; NUFIP2; TAOK1; ABHD15; TP53I13; GIT1; ANKRD13B;	1.5E-08	Yes	Yes

			CORO6; SSH2; EFCAB5; NSRP1; SLC6A4			
12	54302849-54380016	rs9804784	HOXC13; HOXC12; HOXC11; HOXC10; RP11-834C11.12; HOXC6; HOXC9	1.8E-08	Yes	Yes
10	21629890-22501895	rs10828247	CASC10; SKIDA1; MLLT10; DNAJC1; EBLN1	2.5E-08	No	No
15	67470134-68219974	15:67976089_ACT_A	SMAD3; AAGAB; IQCH; C15orf61; MAP2K5; SKOR1	2.7E-08	No	Yes
9	109986827-110131422	rs7026973	RAD23B	2.9E-08	No	No
14	91300612-91615586	rs13379045	RPS6KA5; C14orf159	3E-08	Yes	No
20	45461109-45608564	rs9679828	EYA2	3.2E-08	Yes	Yes
1	11459353-12081637	rs12752879	C1orf167; MTHFR; CLCN6; NPPA; NPPB; KIAA2013; PLOD1; MFN2; MIIP	3.2E-08	No	No
6	101204504-101922321	rs9485491	ASCC3; GRIK2	4E-08	No	No
11	62277701-62523559	rs58720921	AHNAK; EEF1G; MIR3654; TUT1; MTA2; EML3; ROM1; B3GAT3; GANAB; INTS5; RP11-831H9.11; C11orf48; METTL12; C11orf83; UBXN1; LRRN4CL; HNRNPUL2-BSCL2; BSCL2; GNG3; HNRNPUL2; TTC9C; ZBTB3; POLR2G	4.7E-08	Yes	Yes

Supplementary Table S4: Interaction model estimates from standard linear regression for the top UKB interaction locus (rs13389219).

Term	Estimate*	Model-based SE	Model-based P	Robust SE	Robust P
Intercept	-1.56	0.054	9.37E-184	0.0539	7.89E-185
Genotype dose (T allele)	-0.0513	0.00215	7.86E-126	0.00238	2.73E-103
Sex (male)	1.18	0.00332	<1E-300	0.00326	<1E-300
BMI (kg/m^2; centered)	0.0817	3.52E-04	<1E-300	4.47E-04	<1E-300
Age (years)	0.0215	0.00198	1.14E-27	0.00197	8.92E-28
Age-squared	-6.1E-05	1.78E-05	0.000612	1.78E-05	0.000607
Genotyping array	0.0685	0.00349	5.9E-86	0.00352	2.46E-84
PC1	-0.621	0.0795	5.44E-15	0.083	7.14E-14
PC2	0.288	0.0894	0.0013	0.0919	0.00176
PC3	0.0964	0.106	0.362	0.106	0.362
PC4	0.19	0.11	0.085	0.11	0.0863
PC5	-0.119	0.11	0.278	0.11	0.279
PC6	-0.0598	0.144	0.678	0.148	0.686
PC7	-0.13	0.117	0.266	0.117	0.269
PC8	0.216	0.114	0.0575	0.113	0.057
PC9	0.0163	0.115	0.888	0.116	0.888
PC10	-0.0204	0.116	0.86	0.116	0.861
Genotype:sex	0.0564	0.00317	9.28E-71	0.00315	1.61E-71
Genotype:BMI	-4.4E-04	3.31E-04	0.184	4.26E-04	0.301
*Effect estimates are provided in units of standard deviations (s.d.) of inverse-normal transformed WHR.					
